# Causal reasoning with forces

**DOI:** 10.3389/fnhum.2015.00001

**Published:** 2015-01-21

**Authors:** Phillip Wolff, Aron K. Barbey

**Affiliations:** ^1^Department of Psychology, Emory UniversityAtlanta, GA, USA; ^2^Beckman Institute for Advanced Science and Technology, University of Illinois at Urbana-ChampaignUrbana, IL, USA

**Keywords:** causal reasoning, mental simulation, causal models, causal learning, knowledge structures, lexical semantics

## Abstract

Causal composition allows people to generate new causal relations by combining existing causal knowledge. We introduce a new computational model of such reasoning, the *force theory*, which holds that people compose causal relations by simulating the processes that join forces in the world, and compare this theory with the mental model theory (Khemlani et al., 2014) and the causal model theory (Sloman et al., 2009), which explain causal composition on the basis of mental models and structural equations, respectively. In one experiment, the force theory was uniquely able to account for people's ability to compose causal relationships from complex animations of real-world events. In three additional experiments, the force theory did as well as or better than the other two theories in explaining the causal compositions people generated from linguistically presented causal relations. Implications for causal learning and the hierarchical structure of causal knowledge are discussed.

## Causal reasoning with forces

Causal relations can be generated by forming links between non-adjacent entities in a causal chain. The phenomenon is exemplified in ordinary causal transitive reasoning. When told, for example, that A *causes* B and that B *causes* C, people can infer that A *causes* C, or when told, for instance, that *Sanding causes dust* and *Dust causes sneezing*, they conclude that *Sanding causes sneezing*. In transitive reasoning, such as this, the two component relations (e.g., the two CAUSE relations) are the same (Egenhofer, 1994). Interestingly, the process of deriving new causal relations can also occur in chains in which the component relations differ, that is, in chains involving different kinds of causal relations, including relations such as causing, allowing, and preventing. For example, when told that A *causes* B and that B *prevents* C, people may infer that A *prevents* C (Goldvarg and Johnson-Laird, 2001; Barbey and Wolff, 2006, 2007; Sloman et al., 2009; Khemlani et al., 2014), or more concretely, when told *Sanding causes dust, Dust prevents adhesion*, we can infer that *Sanding prevents adhesion*. When causal relations are formed from different kinds of causal relations, the process is not simple transitive reasoning: instead, the reasoning involves a process known as *relation composition*. More formally, given the relations *a* R_i_ *b* and *b* R_j_ *c*, relation composition is the process that determines the relation (R_k_) that holds between *a* and *c* (Ulam, 1990; Egenhofer, 1994). The process is often symbolized by the relation composition symbol, a small circle “◦,” written between the names of the relations being composed.

**Figure 1:**
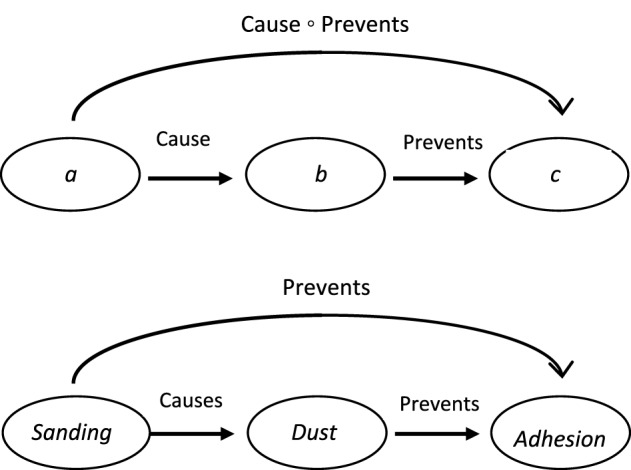
**The top panel shows how relation composition can be realized in a directed graph as a relation between non-adjoining elements in a chain**. The bottom panel replaces the symbols in the top graph with a real world example.

The process of relation composition can be depicted graphically, as shown in Figure 1 (e.g., Rodríguez et al., 2002). In the top panel of Figure 1, the composition of the relation between *a* and *b* and the relation between *b* and *c*, is Cause ° Prevent. In the bottom panel of Figure 1, the symbols in the top graph are fleshed out in an actual example; in this case, the relation composition of CAUSE and PREVENT is PREVENT. Relation composition can occur across a range of relation types. For example, the process applies to relations of equality and inequality: if *a* is equal to *b* and *b* is greater than *c*, then *a* must be greater than *c* (Hein, 2002); temporal relations: if *a* occurs during *b*, and *b* occurs after *c*, then *a* occurs after *c* as well (Allen, 1984; Van Beek, 1989; Hajnicz, 1996; Rodríguez et al., 2002); and spatial relations: if *a* is inside of *b* and *b* is outside of *c*, then *a* must be outside of *c* (Egenhofer, 1994; Rodríguez and Egenhofer, 2000; Skiadopoulos and Koubarakis, 2004).

Recently, researchers have begun to examine relation composition with respect to causation (Sloman et al., 2009; Khemlani et al., 2014), a kind of relation composition that will be referred to as *causal composition*. According to the *mental model theory* (Goldvarg and Johnson-Laird, 2001; Khemlani et al., 2014), causal composition involves operations on relations that specify possible logical entailments, whereas, according to the *causal model theory*, causal composition involves operations on relations that imply families of dependencies (sometimes statistical). According to both of these theories, the process of causal composition occurs over units of cognition that bear little or no resemblance to entities in the outside world, just as the word “tree” bears no resemblance to what it refers to in the actual world. The current state of the literature might be interpreted as indicating that generative processes, such as causal composition, require units of cognition that are inherently abstract in nature and quite different from the codes used in the perception of causal relations. In this paper, however, we describe a new theory of causal composition, the *force theory*, which suggests that the underlying units need not be so abstract. In particular, according to the force theory, relation composition occurs over iconic perceptual codes. The codes are iconic in the sense that they are internally organized in a manner that parallels the structure of the quantities they refer to the physical world. The codes parallel the structure of the physical world just as a road map reflects the spatial organization of the streets and highways in the world. The codes are perceptual in the sense that they are internally organized in a manner that mirrors the internal organization of the mental representations produced from directly perceiving a causal event. The force theory assumes mental representations like those proposed in Kosslyn's (1980) quasi-pictorial theory and Shepard and Chipman's (1970) theory of second-order isomorphisms. Of central interest in this paper will be whether a theory of causal composition based on iconic perceptual codes is able to account for the phenomenon of causal composition as well as, or even better than, theories based on abstract codes. To the extent that this is possible, it would imply that causal reasoning may be based on representations that are perceptual in nature, without the need for abstract mental codes (Barsalou, 1999; Wolff, 2007; Battaglia et al., 2013; Wolff and Shepard, 2013).

## Abstract code theories of causal composition

### Mental model theory

According to the mental model theory (Khemlani et al., 2014), causal relations and their composition can be explained in terms of logical relations. As with the other theories to be discussed, the mental model theory assumes three major kinds of causal relations: CAUSE, ALLOW, and PREVENT. The theory holds that these three kinds of causal relations are associated with different combinations of possible co-occurrences. The sets of possible co-occurrences associated with CAUSE, ALLOW, and PREVENT are shown in the first column of Table 1. For example, a CAUSE relation is associated with a set of co-occurrences in which A is present and B is present, A is absent and B is present, and A is absent and B is absent. Critically, the theory holds that a CAUSE relation is not associated with a co-occurrence in which A is present and B is absent. The theory implies, then, that in a CAUSE relation the presence of A is sufficient for the occurrence of B, but not necessary. ALLOW relations, on the hand, are associated with co-occurrences in which A is present and B is present, A is present and B is absent, and A is absent and B is absent; they are not associated with co-occurrences in which A is absent and B is present. The theory implies, then, that in an ALLOW relation, the presence of A is necessary for the occurrence of B, but not sufficient. Finally, PREVENT relations are associated with co-occurrences patterns in which A is present and B is absent, A is absent and B is present, and A is absent and B is absent, but not a co-occurrence pattern in which A is present and B is present. Negation is handled using the negation operator, ¬. Negating the antecedent or consequent of a causal relation involves flipping the states of affairs of the antecedents and consequents (respectively) in all of the possible co-occurrences.

**Table 1:** **Possible co-occurrences associated with the concepts CAUSE, ALLOW, PREVENT, and various derivatives though negation**.

**Basic relation**			**Antecedent negated**			**Consequent negated**		
CAUSE	a	b	¬A_CAUSE	¬a	b	CAUSE¬B	a	¬b
	¬a	b		a	b		¬a	¬b
	¬a	¬b		a	¬b		¬a	b
ALLOW	a	b	¬A_ALLOW	¬a	b	ALLOW¬B	a	¬b
	a	¬b		¬a	¬b		a	b
	¬a	¬b		a	¬b		¬a	b
PREVENT	a	¬b	¬A_PREVENT	¬a	¬b	PREVENT¬B	a	b
	¬a	b		a	b		¬a	¬b
	¬a	¬b		a	¬b		¬a	b

*¬A, Lack of antecedent, ¬B, Lack of consequent*.

An interesting consequence of the model theory is that it implies certain correspondence relationships between different types of causal expressions. For example, as shown in Table 1, the theory predicts that *A prevents B* and *A causes ¬B* should be paraphrases of each other because they share the same set of co-occurrences and hence the same truth conditions. In support of this particular correspondence, the claim *pain prevents sleep* can be fairly accurately paraphrased as *pain causes lack of sleep* (Wolff et al., 2010). As we will see, correspondences between negated and non-negated expressions of causation play an important role in the interpretation of the findings in several experiments: for example, if a theory predicts that the overall relation for a causal chain is PREVENT, and people respond with CAUSE¬B, their response is not necessarily inconsistent with the theory's prediction since the two expressions are assumed to have the same meaning.

The mental model theory provides an account of the composition of various types of causal relations. In discussing this and the following theories, we will use terminology typical of syllogistic reasoning. That is, we will refer to the relations in the causal chain as “premises” and their composition, as a “conclusion.”

Table 2 outlines a simplified version of the steps involved in deriving a conclusion in the mental model theory (see Khemlani et al., 2014 for details). The first step is to list the possible co-occurrences associated with each causal relation, or premise. For example, in Table 2, the co-occurrences associated with *a causes b* and *b prevents c* are listed. The next main step is to conjoin the sets of possible co-occurrences with respect to their shared common argument. In Table 2, the shared common argument is *b*. Once the sets are conjoined, the common argument (i.e., *b*) can be dropped since the goal of the procedure is to identify the relationship between the unconnected arguments (i.e., *a* and *c*). Any resulting redundancies are dropped as well (e.g., dropping the *b* term results in two a c co-occurrences, so one is deleted). Finally, the kind of relation entailed by the conjoining of the co-occurrences can be interpreted. In Table 2, the co-occurrences (a c), (a c), (a c) make up the set of co-occurrences associated with PREVENT. Thus, according to the mental model theory, when CAUSE is composed with PREVENT, the resulting relation between the non-connected arguments, or conclusion, is PREVENT.

**Table 2:** **Important steps in composing CAUSE and PREVENT relations in the mental model theory**.

**Step1: represent premises**				**Step 2: conjoin premises**			**Step 3: reduce**		**Step 4: interpret**
**A causes B**		**B prevents C**							
a	b	b	¬c	a	b	¬c	a	¬c	*A prevents C*
¬a	b	¬b	c	¬a	b	¬c	¬a	c	
¬a	¬b	¬b	¬c	¬a	¬b	c	¬a	¬c	
		¬a	¬b	¬c					

### Causal model theory

A second symbolic approach to causal composition is represented in Sloman et al. (2009) causal model theory. The causal model theory represents an approach to causal composition based on causal Bayesian networks. In causal Bayesian networks, variables are connected to one another with “arcs,” as in A → B. Each arc is associated with a set of conditional probabilities in conjunction with assumptions about the effect of actual or hypothetical interventions (Woodward, 2003, 2007; Sloman, 2005; Schulz et al., 2007). In the causal model theory, the notions of CAUSE, ALLOW, and PREVENT are represented in terms of different causal structures. When the causal relations are deterministic, it is often convenient to represent variables and arrows of a graph structure in terms of structural equations (Sloman et al., 2009). In the causal model theory, structural equations, just as in structural equation modeling (SEM), serve as a way to identify and think about relationships between variables. For example, the graph A → B can be instantiated in a structural equation such as B:= A (Sloman et al., 2009; see also Hitchcock, 2001). The “:=” relation differs from “equal to” in that variables on one side of the relation cannot be moved to the other: causes must be kept on one side and effects on the other. Loosely stated, “:=” can be likened in meaning to the notion of “gets”; thus, rather than saying “B equals A,” one could say “B gets A.” According to their theory, the claim *A causes B* would be represented by the structural equation B:= A. The concept of ALLOW is associated with a different structural equation; namely, the claim *A allows B* would be represented as B:= A and X, in which the variable X is an accessory variable. In the causal model theory, an accessory variable is an additional causal factor or event that is necessary for the main event, e.g., B, to occur. Accessory variables may either be unknown or given by prior knowledge or given by the environment (Sloman et al., 2009). Sloman et al. (2009) speculate that the concept of PREVENT is vaguer than CAUSE or ALLOW and, as a consequence, may be represented by several structural equations. On their account, the claim *A prevents B* could be represented by either B:= ¬A, B:= ¬A and X, or B:= ¬A and ¬X^1^. Negation is handled using the negation operator. For example, whereas the claim *A causes B* would be represented B:= A, the claim *Not-A causes B* would be represented as B:= ¬A.

To account for relation composition, Sloman et al. (2009) make several processing assumptions. According to one of these assumptions, relations are combined via substitution. Consider, for example, the following relations:

A causes B.
B causes C.

These two relations can be represented as

B:= A.
C:= B.

The theory holds that the representations can be combined with respect to their common argument to obtain, through substitution, C:= A. According to the causal model theory, a structural equation such as C:= A is characteristic of a CAUSE conclusion; hence, people should infer that the composition of two CAUSE relations is another CAUSE relation. A different conclusion is predicted from the composition of a CAUSE and an ALLOW relation. The theory would represent the claims *A causes B, B allows C*, with the structural equations: B:= A and C:= B and X. Substituting the first “B” into the second equation would lead to C:= A and X. According to the theory, such an equation would be indicative of an ALLOW relation; hence, the theory predicts that the composition of CAUSE and ALLOW would be an ALLOW relation.

For certain relation compositions, the causal model theory, unlike the mental model theory, predicts more than one conclusion. In particular, for compositions involving two PREVENT relations, the causal model theory predicts either CAUSE or ALLOW, depending on whether or not the PREVENT relations include an accessory variable.

Negation is handled using the negation operator. For example, while the claim *A causes B* would be represented B:= A, the claim *Not-A causes B* would be represented as B:= ¬A. Interestingly, the structural equation for *Not-A causes B* is the same as one of the equations associated with PREVENT, since the claim *A prevents B* can be represented as B:= ¬A. Absent effects are handled by negation as well. For example, the claim *A causes not-B* would be represented as ¬B:= A.

## A perceptual iconic theory of causal composition

### The force theory

In this paper we introduce a new theory of causal composition, the force theory. The force theory is based on Talmy's (1988) theory of force dynamics. In the force theory, individual interactions involve two main entities: an *affector* and a *patient* (the entity acted on by the affector). The theory holds that people specify causal relations in terms of configurations of forces that are evaluated with respect to an endstate. It is assumed that the configurations of forces associated with different kinds of causal relations can be directly aligned with the configuration of forces present in various domains. In particular, it is assumed that people specify the origin and direction of the various forces in a configuration of forces, and to a limited extent, the magnitudes of these forces. This alignment makes the representation of causation in the force theory iconic. An endstate can be conceptualized as a location in a space that the patient either moves toward or away from. The forces may be physical, psychological (e.g., intentions), or social (e.g., peer pressure) (Talmy, 1988; Wolff, 2007; Copley and Harley, 2014). It is assumed that people are able to conduct partial reenactments of the processes that join forces in the world (see Wolff and Zettergren, 2002; Wolff, 2007; White, 2011, 2012; Hubbard, 2013b,c). A reenactment involves specifying the objects and the forces acting on those objects as well as carrying out a simulation. A simulation involves performing a qualitative type of vector addition as specified and empirically examined in Wolff's (2007) Experiment 4 (see also Hubbard, 2010). The forces in these simulations are assumed to be underspecified and often inaccurate, especially with respect to magnitude (Wolff, 2007). As discussed in Hubbard (2005, 2012), people's notions of force may often be incorrect, but good enough to be adaptive. It is also assumed that people may often infer forces when they are not actually present, as when they infer forces in the context of chance occurrences (e.g., closing a book and the lights going out) or when watching a magic show (Wolff and Shepard, 2013).

### Representing individual causal relations

The force theory predicts that there should be three main causal concepts, CAUSE, HELP, and PREVENT. These three concepts can be differentiated with respect to three dimensions: (a) the *tendency* of the patient for an endstate, (b) the presence or absence of *concordance* between the affector and the patient, and (c) *progress toward the endstate* (essentially, whether or not the result occurs). Table 3 summarizes how these dimensions differentiate the concepts of CAUSE, HELP (also ALLOW and ENABLE), and PREVENT. When we say, for example, *High winds caused the tree to fall*, we mean that the patient (the tree) had no tendency to fall (Tendency = No), the affector (the wind) acted against the patient (Concordance = No), and the result (falling) occurred (Endstate approached = Yes).

**Table 3:** **Representations of several causal concepts**.

	**Patient tendency for endstate**	**Affector-patient concordance**	**Endstate approached**
CAUSE	No	No	Yes
HELP (also ALLOW and ENABLE)	Yes	Yes	Yes
PREVENT	Yes	No	No

**Figure 2:**
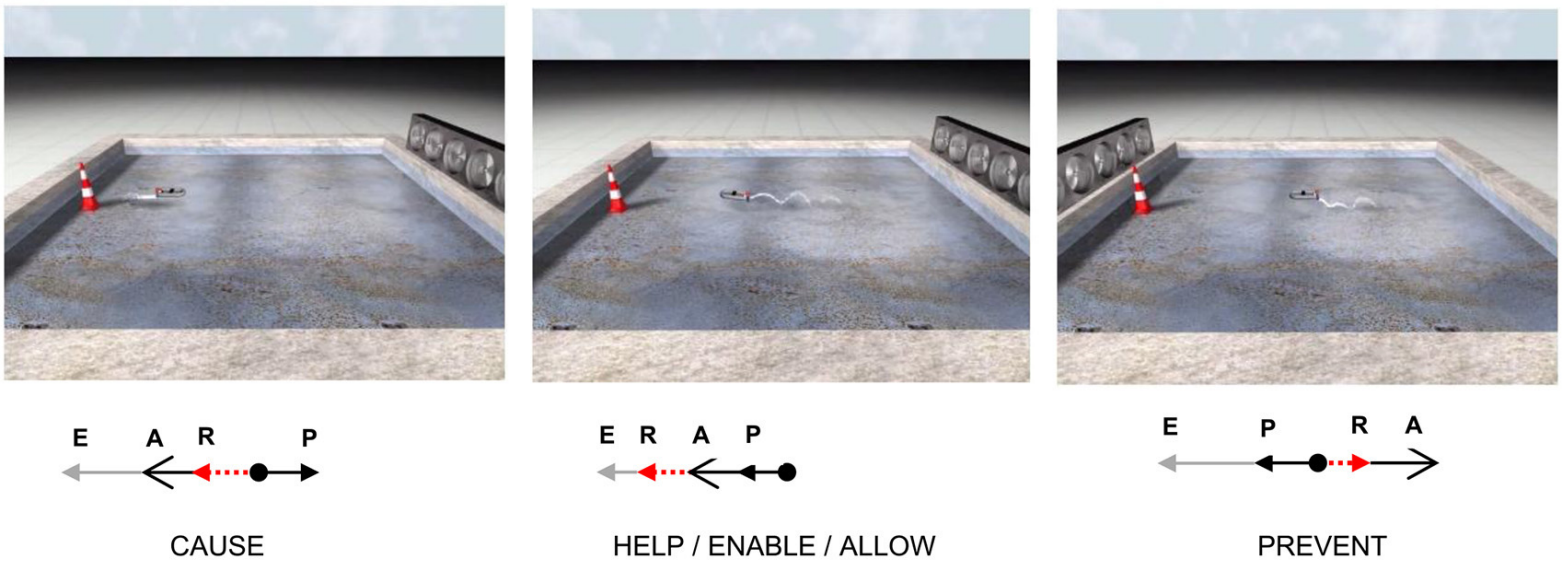
**The images above each depict a scene showing a boat, a bank of fans, and a cone**. In the CAUSE scene, the boat motors away from the cone, but is pushed back to the cone by the fans. In the HELP scene, the boat motors toward the cone, and the fans push it along in the same direction. In the PREVENT scene, the boat motors toward the cone, but the fans push it back away from the cone. Free-body diagrams associated with each type of causation are shown below each scene. In these diagrams, **A**, the affector force, **P**, the patient force, **R**, the resultant force, and **E**, endstate vector, which is a position vector, not a force.

The force theory specifies how the notions of tendency, concordance, and progress toward the endstate can be instantiated in non-linguistic terms, namely in terms of force and position vectors. The way the theory does this is shown in Figure 2. Each scene in Figure 2 shows a situation involving a pool of water, a boat with an outboard engine, a bank of fans, and a buoy. Below each scene is a free-body diagram which makes explicit the direction and relative magnitude of the forces in each scene.

As is customary in the construction of free-body diagrams in physics, the forces are shown acting on only one object, the patient; the free-body diagrams do not show the locations of the affector (i.e., **A**), only the direction and magnitude of the affector's force on the patient. In each of the configurations shown in Figure 2, the patient is associated with a force (i.e., **P**). The force associated with the patient, **P**, can be generated in a number of ways, including from gravity or mechanisms internal to the patient, or from the patient's resistance to changes in speed or direction due to frictional forces or momentum (Wolff, 2007). Copley and Harley (2014) provide a compelling argument for the view that the force associated with the patient is often best understood as emerging from the patient's position in a “normal field.” In their account, the normal field gives rise to an object's tendency to fall due to gravity, as well as more abstract tendencies such as an entity's tendency to “grow,” “redden” or “straighten.” In Figure 2, the patient's force corresponds to the force generated by the boat's motor.

According to the force theory, the patient has a tendency for the end-state when the patient vector, **P**, points in the same direction as the end-state vector, **E**; otherwise, **P** points in a different direction. The patient's tendency for a particular endstate is similar to the Bayesian notion of a prior probability because it expresses what will happen before other factors are taken into account. It differs from being a prior probability in that it does not express an uncertainty. Returning to the force theory, the patient and the affector are in concordance when their respective vectors point in the same direction. Finally, a patient entity will approach the end-state when the resultant (sum) of the **A** and **P** vectors, **R**, is in the same direction as the end-state vector, **E**. Importantly, the end-state vector, **E**, is a position vector, not a direction vector. Hence, the length of the end-state vector specifies how close the patient is to reaching the end-state.

Support for the force theory's account of CAUSE, ALLOW, and PREVENT was provided in a series of experiments in which participants categorized 3-D animations of realistically rendered objects with trajectories that were wholly determined by the force vectors entered into a physics simulator. As reported in Wolff (Wolff and Zettergren, 2002; Wolff, 2007), people's descriptions of the events closely matched the predictions of the model.

### Combining relations in the force theory

In addition to explaining the representation of individual causal relations, the force theory also explains how individual relations can be joined to form causal chains and how these chains may then be used to derive new overarching causal relations. In the force theory, a new overarching configuration of forces is derived by selecting and adding certain forces from the sub-configurations. Specifically, an affector force is determined by selecting the affector force from the first sub-configuration of forces; a patient force is derived from the vector addition of all of the patient forces in the sub-configurations; and an endstate vector is determined by selecting the endstate vector from the last sub-configuration of forces. While the process used for deriving an overarching configuration of forces is the same across all types of causal chains, the manner in which the causal chains is constructed in the first place depends on whether the chain involves the transmission or removal of forces.

**Figure 3:**
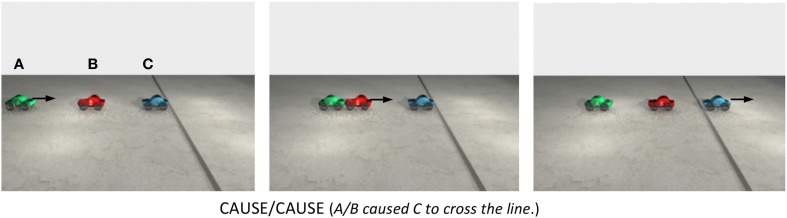
**The animation instantiates a CAUSE/CAUSE causal chain in which A causes B, and B causes C**. A begins moving first. It hits B, sending B into C, which then moves over the line. The arrows indicate the direction of the cars' motion. The animation can be summarized by the sentence *A caused C to cross the line*.

In transmissions of force, the resultant of the first sub-configuration of forces is used as the affector of the subsequent configuration of forces. The idea can be explained in terms of a simple sequence of collision events like the one depicted in Figure 3. In Figure 3, car A begins moving first, it hits B, and B then hits C, sending C over the line. The animation can be summarized by the sentence *A caused C to cross the line*, implying that the individual relations in the causal chain can be combined to form an overarching causal relationship between non-contiguous entities^2^.

**Figure 4:**
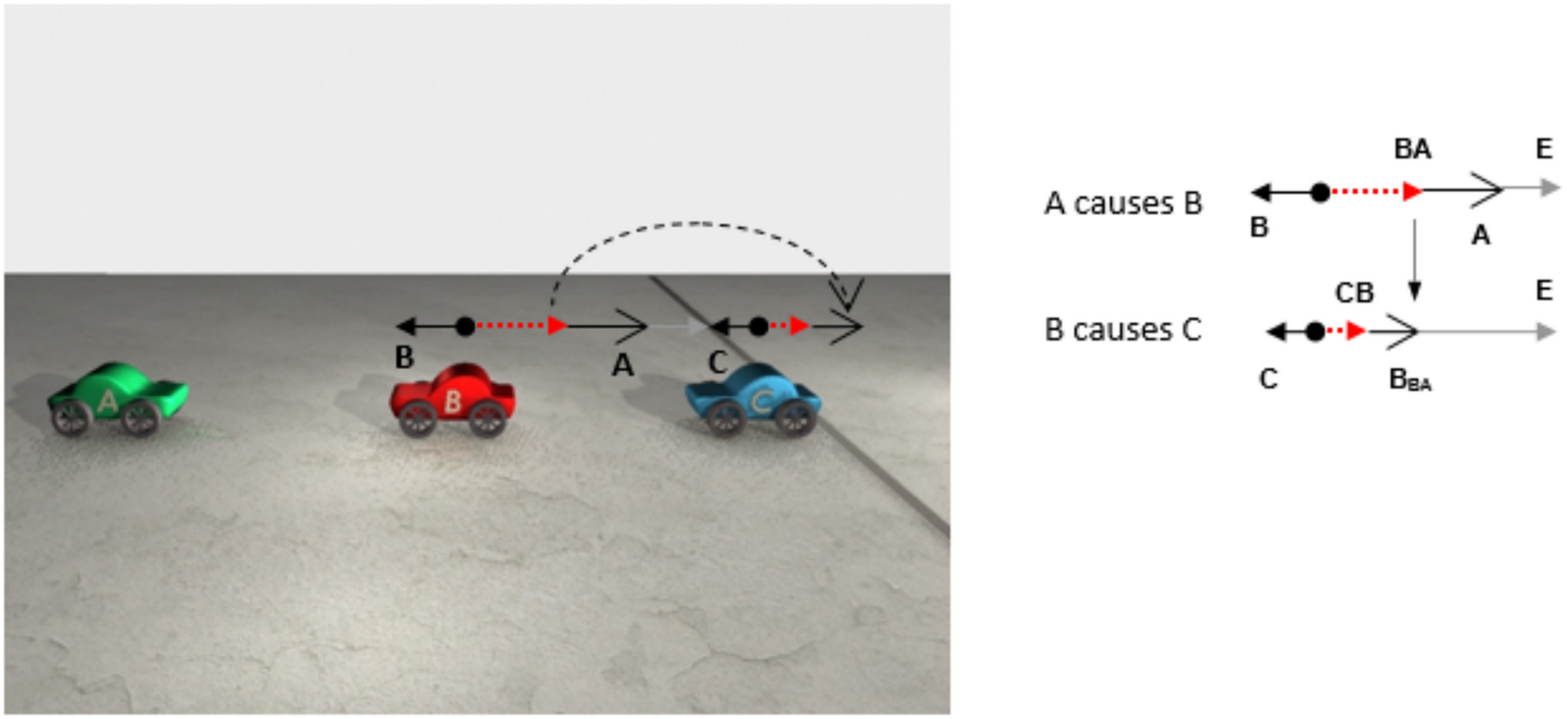
**Above cars B and C are CAUSE configurations of forces**. The smaller vectors pointing to the left are the patient vectors acting on cars B and C (i.e., friction). The longer vectors (with big heads) pointing to the right are affector vectors and the dashed vectors are the resultant vectors. In this sequence of collisions, the resultant vector associated with car B becomes the affector vector acting on car C. On the right side are two free-body diagrams depicting the same configurations of forces shown on the left, but this time, they are arranged vertically rather than horizontally. In the free-body diagrams, the line pointing down shows how the resultant of the first CAUSE configuration becomes the affector in the second CAUSE configuration.

Figure 4 offers a different view of the same sequence of events shown in Figure 3. On the left side of Figure 4 is a picture of the first frame of the animation. Above car B and C are CAUSE configurations of forces. In the first CAUSE configuration, the affector force (**A**) comes from car A and the patient force (**B**) comes from car B. In the second CAUSE configuration, the affector force is the resultant of the **A** and **B** forces in the first CAUSE configuration. The patient force in the second CAUSE configuration is **C**, specifically, its resistance to moving forward. The resultant of the affector and patient forces acting on the third car sends it over the line. In Figure 4, the direction and speed of A and B are determined by the direction and magnitude of the (red) affector vectors acting on A and B.

**Figure 5:**
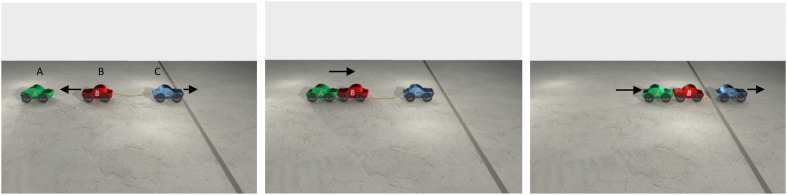
**The still frames depict key stages in a PREVENT/PREVENT chain**. First, C attempts to cross the line but is prevented by B. Then, A pushes B toward C, preventing B from preventing C. With the removal of B's preventive force, C crosses the line. The animation can be summarized by the sentence *A allowed C to cross the line*.

Whereas some causal chains involve transmissions of force, other causal chains involve the removal of a force. When a chain involves the removal of a force, the manner in which resultant forces become affector forces reverses. To illustrate, consider, a chain of PREVENT relations such as A PREVENTS B and B PREVENTS C. A chain of PREVENT relations is known as a double prevention (Collins, 2000; Hall, 2000, 2004, 2006; Schaffer, 2000; Dowe, 2001; McGrath, 2005; Sartorio, 2005; Livengood and Machery, 2007; Lombrozo, 2010; Wolff et al., 2010; Walsh and Sloman, 2011). At first glance, it may be unclear how a double prevention could be realized because if A prevents B, B cannot then prevent C because B has already been prevented. The solution to how a double prevention can occur rests in the order in which the prevent relations are realized. In particular, a double prevention can occur if the second prevent relation is realized before the first prevent relation is realized. So, for example, if A prevents B and B prevents C, such a chain can occur if B is first preventing C and then A prevents B. The intuition behind this can be illustrated with a real world example. Consider pulling a plug to allow water to flow down a drain. Such a sequence of PREVENTs begin with the plug (B) preventing the water (C) from draining (that is the second relation in the double prevention). Then, someone (A) prevents B by pulling the plug, that is, by removing B's force on C. Note that when A pulls B, A opposes not just the force associated with B, but also the force associated with C, that is, the resultant of the **B** and **C** forces (the plug and the water). Thus, in the case of double prevention, the resultant of the second premise (**CB**), which is computed first, serves as the patient vector in the first premise (**B_CB_**). The way forces are transmitted in a double prevention can be illustrated in a different way based on the chain depicted in Figure 5.

**Figure 6:**
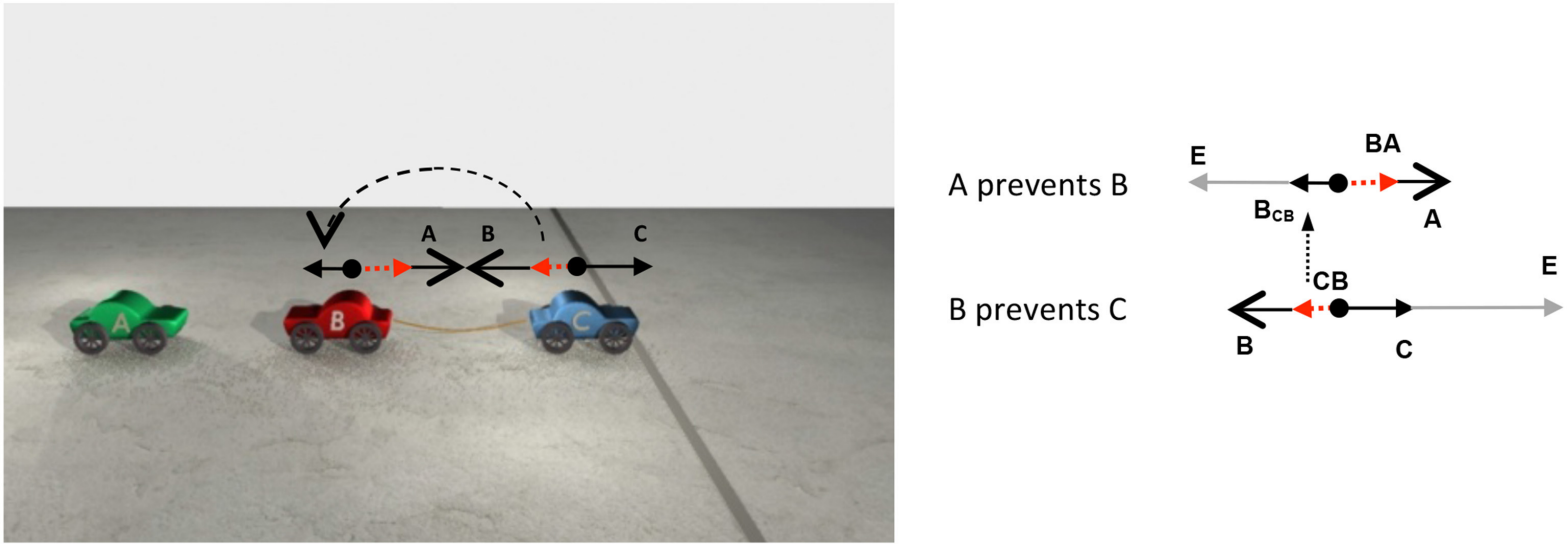
**The scene depicts the configuration of forces instantiated in a PREVENT/PREVENT causal chain**. The smaller vectors pointing left and right are patient vectors, while the longer vectors (with big heads) are affector vectors. The dashed vectors are resultant vectors. On the right side are two free-body diagrams depicting the same forces shown on the left arranged vertically. In the diagrams, the vector **E** is the position vector pointing to the end-state, which, in the animation on the left, is the area on the right side of the line. Note that in a double prevention, the resultant vector of **B** and **C**, **CB**, becomes the patient vector in the interaction between cars B and A.

In the beginning of the animation depicted in Figure 5, C approaches the line. B prevents C from crossing the line by pulling C back with a rope. The middle panel shows A pushing B back, thereby loosening the rope between B and C. In the panel on the far right, with the removal of B's force on C, C crosses the line. The animation can be summarized by the sentence *A allowed C to cross the line*, implying that the individual relations in the sequences of events can be combined to form an overarching causal relationship between non-contiguous entities. A different way of viewing the same sequence of events is shown in Figure 6.

On the left side of Figure 6 is a frame from one of the middle frames of the animation. Above B and C are PREVENT configurations of forces. On the left side of Figure 6, in the second PREVENT configuration (involving car C), the affector force comes from car B and the patient force comes from car C. In the first PREVENT configuration (on the left side of Figure 6), the affector force comes from A while the patient force comes from the sum of the forces **B** and **C**, that is, **BC**.

On the right side of Figure 6 is a pair of free-body diagrams depicting the same configuration of forces shown in the frame of the animation, this time arranged vertically. The diagram above depicts the configuration of forces acting on car B, while the diagram below depicts the configuration of forces acting on car C. As discussed above, in chains of PREVENT relations, the resultant of the second PREVENT configuration serves as the patient vector in the first PREVENT configuration. This transmission of forces is reflected in the vertical arrow connecting the resultant vector in the lower configuration with the patient vector in the higher configuration.

### Composing causal relations

**Figure 7:**
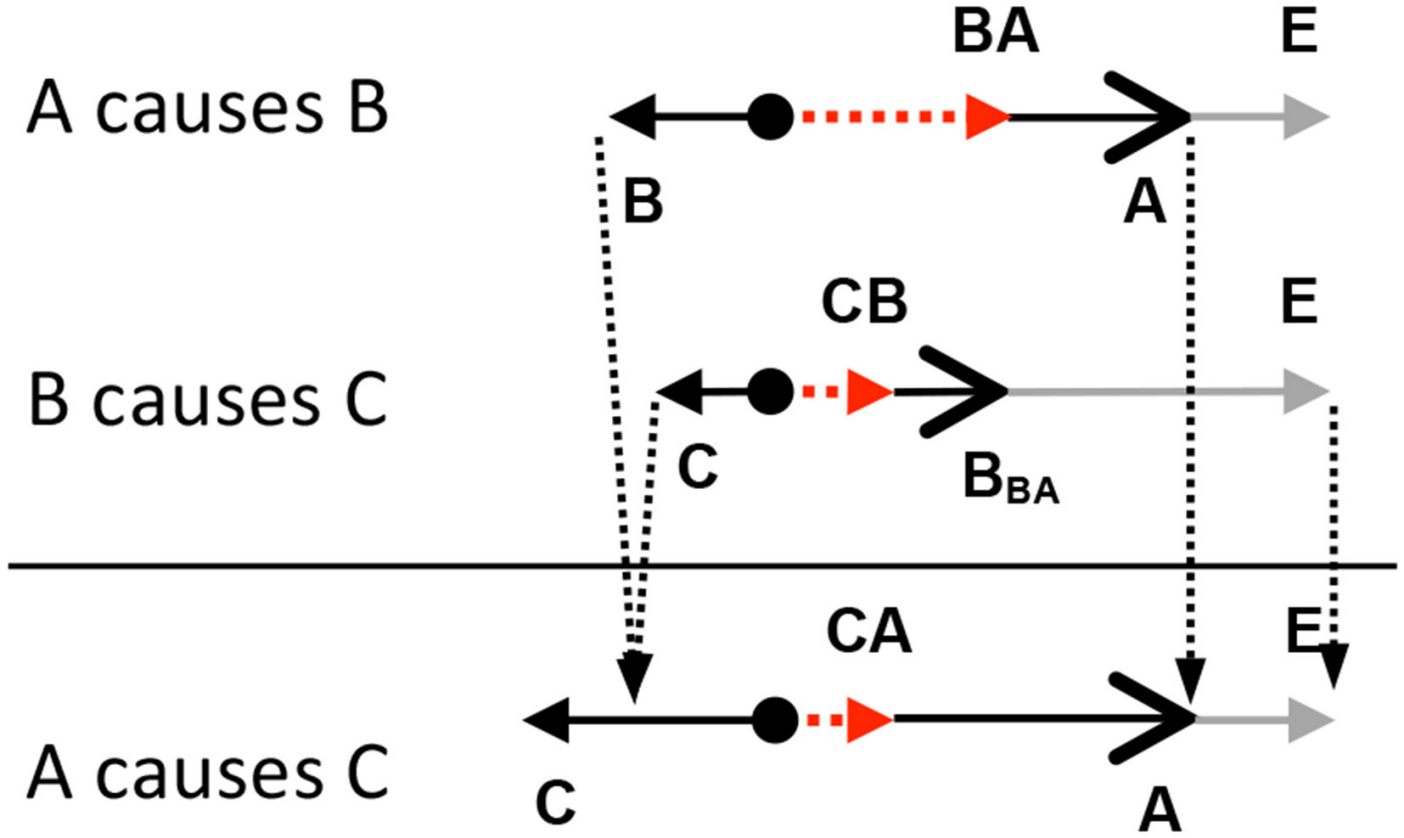
**The affector force in the summary configuration, A, is the affector force in the first relation, A**. The end-state in the summary configuration is the end-state vector from the last relation. The patient force in the summary configuration, **C**, is based on the vector addition of all of the patient forces in the chain (**B** and **C)**.

As discussed earlier, the process of composing causal relations involves constructing an overall summative configuration of forces based on all of the configurations in the chain. Whether the chain involves the transfer or removal of a force, the manner in which a summary configuration is derived remains the same. Figure 7 shows how a summary configuration is derived from a chain of two CAUSE relations. As depicted in Figure 7, the affector in the summary configuration is the affector from the first relation (**A**); the end-state is based on the end-state vector in the last relation (**E**); and the patient in the summary configuration is derived by summing all of the remaining forces in the causal chain (**B** + **C**).

### Allow relations

The force theory offers an account of how people represent ALLOW relations. Following McGrath (2003), we propose that ALLOW relations are necessarily based on double preventions (see also Barbey and Wolff, 2007; Wolff et al., 2010). In the simplest case, ALLOW relations involve removing a force that was originally preventing an event from happening. An example is given in the animation depicted in Figure 5: when A pushes B toward C, it allows C to cross the line. This account of ALLOW was supported in a set of studies described in Wolff et al. (2010). Participants saw animations like the one shown in Figure 5 and were asked to choose one of several possible descriptions. Across a range of double preventions, participants indicated that the relationship between the first and last entities in a double prevention instantiated an ALLOW relation. We further propose that the concepts of ENABLE and LET are closely related to that of ALLOW. All three concepts entail double preventions but differ slightly in the manner in which the particular PREVENT relations are instantiated^3^. In all cases of double prevention, the chaining of two PREVENT relations leads to what could be called a positive relation, much like double negatives in a sentence imply a positive.

### Accounting for negation

The force theory offers an account of the negation of causal relations. There are two kinds of causal negation, one of which is causation by omission. Causation by omission is causation in which an absence of an influence brings about an effect, as in *The absence of nicotine causes withdrawal* or *Not watering the plant caused it to wilt* (Schaffer, 2000; Wolff et al., 2010). Causation of omission can be expressed by a not-operator applied to the first argument of a cause relation, as in ¬A CAUSES B. In this paper, we refer to a causal relation like ¬A CAUSES B with the expressions ¬CAUSE or ¬C. Following McGrath (2003), we propose that causation by omission is another way of describing a double-prevention. When we say A PREVENTS B and B PREVENTS C, we can re-express the double prevention as causation by omission: the lack of B allows/causes C. In a series of studies in Wolff et al. (2010), we found evidence for this proposal: when shown animations of double preventions, people endorsed statements stating that the lack of the second entity (i.e., B) in a double prevention allowed or caused the occurrence of the third entity. In particular, when people were shown double preventions like the one shown in Figure 6, they were willing to endorse the statement *the absence of B caused/allowed C to cross the line*. With respect to the force theory, an expression like ¬A CAUSES B entails the occurrence of a double prevention before the CAUSE relation, thus leading to a three-premise chain, that is, A PREVENTS B, B PREVENTS C, and C CAUSES D. A conclusion is generated between the A and D entities like any other relation composition. This conclusion is then re-phrased in terms of the absence of the second entity, i.e., *Lack of B caused D* (Wolff et al., 2010). It should be noted that in causation resulting from an omission, force is not transmitted from the cause to the effect; rather, a force is removed or not realized and an effect results (Wolff et al., 2010).

A second kind of causal negation is the causation of an absence, as exemplified in expressions of the form A CAUSES ¬B, or more intuitively, by such statements as *Pain causes lack of sleep* and *Black holes allow no escape*. We propose that people represent causation of an absence by treating the negation of the consequent as a PREVENT relation in the causal chain. The PREVENT relation is added to the causal chain by assuming an unnamed entity—which can be referred to by *x—*to connect the relations. Expressions of the form *A* CAUSES ¬B are thereby represented as A CAUSES x, x PREVENTS B. The overarching relation implied by this causal chain is based on the relation composition of CAUSE and PREVENT relations, which according to the force theory, is a PREVENT relation virtually 100% of the time. Thus, according to the force theory A CAUSES ¬B is virtually synonymous with A PREVENTS B^4^.

### Accounting for multiple conclusions

**Figure 8:**
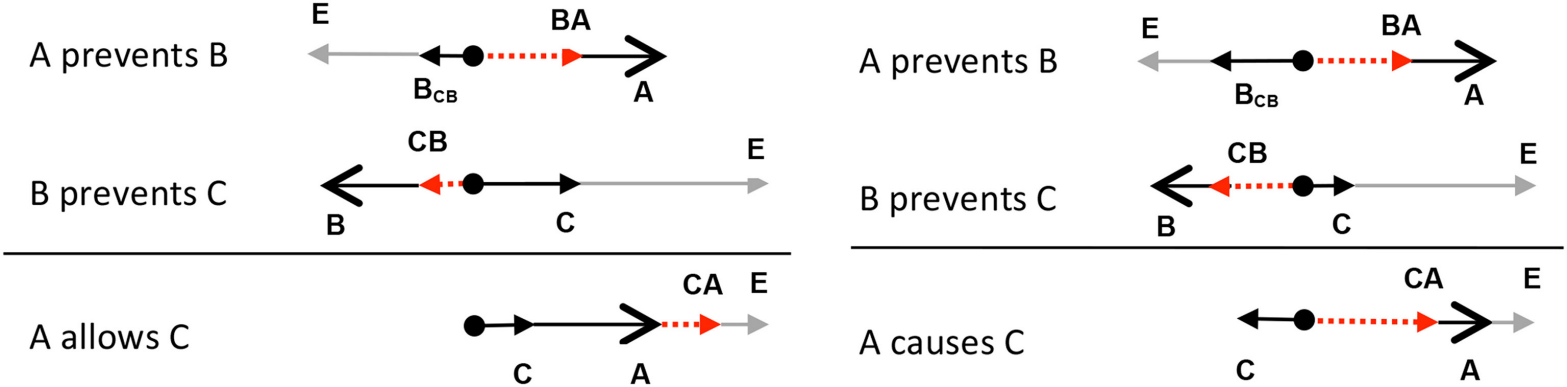
**The composition of two PREVENT relations can either lead to an ALLOW (left side) or CAUSE (right side) conclusion**.

As discussed in the previous sections, the composition of a double prevention can lead to an ALLOW conclusion. However, interestingly, the composition of a double prevention sometimes leads to a CAUSE conclusion (McGrath, 2003; Barbey and Wolff, 2006, 2007; Chaigneau and Barbey, 2008; Sloman et al., 2009; Wolff et al., 2010). Consider, for example, the double prevention instantiated in dropping a pencil to the floor: initially, a person prevents the pencil from falling by holding it in his or her hand, but this prevention is prevented by opening the hand, with the pencil falling as a result. One could describe this situation as instantiating an ALLOW relation: *The person allowed the pencil to fall to the floor*, but one could also describe the situation as instantiating a CAUSE relation: *The person caused the pencil to fall to the floor*. Some scenarios seem to be biased more toward ALLOW interpretations, such allowing water to flow down the drain by pulling a plug. Other scenarios are more biased toward CAUSE interpretations. Imagine a situation in which a person knocks out a post resulting in the collapse of a roof. It sounds more natural to say the person caused the roof to collapse than that he allowed the roof to collapse. The question of what biases a double prevention toward CAUSE or ALLOW is interesting, but for present purposes, we point out that the force theory predicts that double preventions are potentially ambiguous between CAUSE and ALLOW relations. The reason why is highlighted exemplified by the two double prevention shown in Figure 8.

As shown on the left side of Figure 8, in some double preventions, adding the two patient vectors in the premises results in a patient vector in the conclusion (**C**) that points toward the endstate, leading to an ALLOW configuration. On the other hand, as shown on the right side of Figure 8, adding the patient vectors can also result in a patient in the conclusion pointing away from the endstate, resulting in a CAUSE configuration. Although certain sets of premises may be compatible with more than one conclusion, the force theory makes it possible to determine the probability of any conclusion for a given set of premises. One way in which these probabilities can be calculated is by using integral calculus, as explained in Appendix B of the Supplemental Materials. Another way is to use an iterative procedure that varies the magnitudes of each of the forces in the causal chain and then counts the number of times a particular conclusion is generated. A program has been written to conduct such a process (http://psychology.emory.edu/cognition/wolff/software.html). The results from this simulation (as well as from integral calculus) indicate that double preventions will lead to ALLOW conclusions 62% of the time, and CAUSE conclusions 38% of the time. In other relation compositions, only one conclusion is predicted. For example, the composition CAUSE◦PREVENT is predicted to give rise to PREVENT conclusions 100% of the time. In still other relation compositions, the theory still predicts only one conclusion, but at a weaker level. For example, the composition PREVENT◦CAUSE gives rise to PREVENT conclusions 37% of the time. The remaining 63% of the conclusions are associated with an undefined configuration of forces. Under these conditions, we predict that people would associate a PREVENT◦CAUSE composition with a PREVENT conclusion, but to a weaker degree than they would a CAUSE◦PREVENT composition, which result in PREVENT conclusions 100% of the time. Thus, in the absence of clear information about the magnitude of the vectors, the force theory explains how a particular causal chain may be probabilistically related to a particular conclusion. In effect, the force theory explains how different causal structures can give rise to different probability distributions for different conclusions.

## Summary and predictions of the theories

As discussed in the previous sections, three theories provide accounts of how people compose causal relations. Each of these theories makes assumptions about the basic units of cognition that are involved in causal reasoning, the complexes of units that define causal relations, the processes involved in causal composition, and the number of conclusions that may follow from a particular causal composition. These assumptions are summarized in Table 4.

**Table 4:** **Three models of causal composition and how they compare on several key dimensions**.

	**Mental model theory**	**Causal model theory**	**Force theory**
Basic units of cognition	States of affairs	Variable/event states	Forces
Basic relations between units	Co-occurrence	Functional relation	Spatial orientation/Relative magnitude
Complexes of units defining causal relations	Mental models	Structural equations	Configurations of force
Process involved in relation composition	Conjoining mental models	Variable substitution	Vector addition
Number of conclusions per composition	One	Sometimes more than one	Sometimes more than one

One of the key differences between the three theories concerns whether they predict causal composition can give rise to multiple conclusions. The mental model theory predicts a single conclusion for each composition, whereas the causal model theory and the force theory predict that certain causal compositions can give rise to multiple conclusions. The indeterminacy predicted by the force theory may come as a surprise given the theory's grounding in perceptual experience. The indeterminacy predicted by the force theory does not simply emerge with the shift to multiple events: in the force theory, a particular kind of causal relation (e.g., CAUSE) viewed multiple times will give rise to a set of causal relations of the same kind (e.g., CAUSE). Rather, the indeterminacy predicted by the force theory emerges with a shift from single relations to causal chains viewed multiple times: the theory predicts that relation compositions conducted over the same kind of causal chain multiple times will sometimes give rise to different kinds of overarching causal relations. In effect, the force theory offers an account of how deterministic causation might be related to probabilistic causation.

Arguably, the most important difference between the three theories concerns the basic units of cognition. In the case of the mental model and causal model theories, the underlying unit complexes are abstract. In the case of the force theory, in contrast, the underlying units are iconic. The iconic nature of the mental units in the force theory allow it to make predictions that the other theories cannot make. In particular, the force theory is able to make predictions about how causal chains and their negations may be instantiated in the physical world. This is potentially important because if the force theory is right, then the mental codes used in the perception of causal events may be the same codes used in causal reasoning.

### Experiment 1

In the following experiment we examined whether the configurations of forces specified in the force model can be entered into a physical simulator to produce animations reflecting real-world causal chains and then whether these animations are identified by people as instantiating the kinds of causal relations specified in the theory. These predictions were tested by creating the nine possible kinds of causal chains that can be formed from all possible pairings of CAUSE, ALLOW, and PREVENT. The nine types of animations used in this experiment are listed in Table 5, two of which were depicted in Figures 3, 5. The force theory not only predicts how different kinds of causal chains might be instantiated in the physical world, but also how these causal chains might be described. The mental model and causal theories are unable to predict how various kinds of causal chains might be instantiated, but are able to predict how these chains might be described once the chains are represented in terms recognized by the theories. The predictions of the three theories are shown in Table 5.

**Table 5:** **Predicted relation composition for each type of causal chain animation used in Experiment 1**.

	**A/A**	**A/C**	**A/P**	**C/A**	**C/C**	**C/P**	**P/A**	**P/C**	**P/P**
Mental model theory	A	A	P	A	C	P	P	P	P
Causal model theory	A	A	P	A	C	P	P	P	A (50%) C (50%)
Force theory	A	A (76%) C (24%)	P	A (76%) C (24%)	C	P	P	P	A (62%) C (38%)

C, “Cause”; A, “Allow”; P, “Prevent.”

As can be seen in Table 5, for this particular set of relation compositions, the three theories make very similar predictions about how the various kinds of causal chains might be composed, though there are a few differences. For several of the compositions, the force theory predicts more than one conclusion; however, in each case, one conclusion is predicted to dominate. The causal model theory also predicted more than one response for one of the causal chains, P/P, that is for double prevention. However, while the causal model theory predicts that double preventions can lead to either CAUSE or ALLOW conclusions, it does not specify which of these responses should dominate. Given that the causal model is silent with respect to which responses should dominate, we assigned to the theory the prediction that ALLOW and CAUSE relations should each be expected to appear 50% of the time.

There were two sub-experiments. The purpose of Experiment 1a was to verify that the causal chains used in the following Experiment 1b contained the intended component relations. The purpose of Experiment 1b was to test whether participants combined the sub-relations as predicted by the three theories.

### Experiment 1a

In this experiment, each of the animations used in the following experiment, Experiment 1b, were re-rendered as two separate animations: one showing the cars involved in the first causal relation and the other showing the cars used in the second causal relation. For example, in a CAUSE/CAUSE causal chain, participants saw an animation showing either cars A and B or cars B and C. After watching an animation, participants choose the expression that best described the relation between the cars in the animation (e.g., A and B or B and C). The expressions named two cars joined by the verbs *cause, allow*, or *prevent*, or the option “none of the above.” The choices predicted by the force theory are shown in the bottom half of Table 6.

**Table 6:** **Experiments 1a predictions and results for entire chains [Mean (SD)] and Experiment 1b predictions and results individual component relations [Mean (SD)]**.

**Composition**	**A/A**	**A/C**	**A/P**	**C/A**	**C/C**	**C/P**	**P/A**	**P/C**	**P/P**
Prediction	**A**	**A**	**P**	**A**	**C**	**P**	**P**	**P**	**C** (62%) **A** (38%)
“Cause”	0.04 (0.204)	0.08 (0.282)	0.04 (0.204)	0.17 (0.381)	**0.79** (0.415)	0.08 (0.282)	0.04 (0.204)	−	**0.50** (0.511)
“Allow”	**0.63** (0.495)	**0.67** (0.482)	0.13 (0.338)	**0.67** (0.482)	0.13 (0.338)	−	0.08 (0.282)	0.04 (0.204)	**0.50** (0.511)
“Prevent”	0.13 (0.338)	0.04 (0.204)	**0.50** (0.511)	0.04 (0.204)	−	**0.83** (0.381)	**0.79** (0.415)	**0**.**88** (0.338)	−
“No verb”	0.21 (0.415)	0.21 (0.415)	0.33 (0.482)	0.13 (0.338)	0.08 (0.282)	0.08 (0.282)	0.08 (0.282)	0.08 (0.282)	−
**1ST RELATION**									
Prediction	**A**	**A**	**A**	**C**	**C**	**C**	**P**	**P**	**P**
“Cause”	0.04 (0.204)	0.04 (0.204)	0.13 (0.338)	**0.42** (0.504)	**0.88** (0.338)	**0.92** (0.282)	0.17 (0.381)	−	0.08 (0.282)
“Allow”	**0.71** (0.464)	**0.79** (0.415)	**0.75** (0.442)	0.25 (0.442)	0.04 (0.204)	−	0.08 (0.282)	0.08 (0.282)	0.08 (0.282)
“Prevent”	0.08 (0.282)	−	−	0.38 (0.495)	−	0.04 (0.204)	**0.67** (0.482)	**0.79** (0.415)	**0.79** (0.415)
“No verb”	0.17 (0.381)	0.17 (0.381)	0.13 (0.338)	0.21 (0.415)	0.08 (0.282)	0.04 (0.204)	0.08 (0.282)	0.04 (0.204)	0.04 (0.204)
**2ND RELATION**									
Prediction	**A**	**C**	**P**	**A**	**C**	**P**	**A**	**C**	**P**
“Cause”	0.04 (0.204)	**0.50** (0.511)	−	0.08 (0.282)	**1** (0)	−	0.04 (0.204)	**0.83** (0.381)	−
“Allow”	**0.79** (0.415)	−	−	**0.83** (0.381)	−	−	**0.54** (0.509)	0.04 (0.204)	−
“Prevent”	0.04 (0.204)	0.13 (0.338)	**0.96** (0.204)	0.04 (0.204)	−	**0.96** (0.204)	0.08 (0.282)	0.04 (0.204)	**0.96** (0.204)
“No verb”	0.13 (0.338)	0.38 (0.495)	0.04 (0.204)	0.04 (0.204)	−	0.04 (0.204)	0.33 (0.482)	0.08 (0.282)	0.04 (0.204)

*Participant's modal responses are in bold*.

#### Methods

***Participants***. The participants were 24 Emory University undergraduates who took part in the study for course credit. All participants were native speakers of English. In this and all following experiments, participants gave informed consent in accordance with the requirements of the Internal Review Board at Emory University.

***Materials***. Eighteen 3D animations were made from an animation package called Autodesk 3 3ds Max 8. The eighteen animations depicted either the first or second part of one of nine causal chains: A/A, A/C, A/P, C/A, C/C, C/P, P/A, P/C, and P/P. The direction and speed of the cars was calculated using a physics simulator called Havok Reactor, which is a sub-program in 3ds Max. The mass of each car, including its wheels, was 5 kg (approximately 11 pounds). Table A1 in the Supplementary Materials shows of the forces in Newtons entered into the physics simulator for each of the cars that appeared in the animations. As a matter of comparison, the amount of force exerted by gravity on an object with a mass of 1 kg near the surface of the earth is approximately 10 N. The number of cars differed across the animations because the different kinds of relation compositions required different numbers of forces. For example, a C/C chain only requires three cars in order to instantiate three unique forces (see Figures 3, 4), whereas a C/A chain requires four cars in order to instantiate four unique forces because the ALLOW relation is composed of two PREVENT relations. All of the interactions occurred within a single dimension; as a consequence, the directions of the forces listed in Table A1 are described as either to the right or left. In this simulated world, the cars were 2 feet, 5 inches. The camera was directed toward the center of the interaction at an angle of 15 degrees and was located 18 feet, 8 inches away from the center of action. In every animation, A was green, B was red, C was blue, D was yellow, and E was purple. The cars moved over a gray cement surface and the sky was a slightly lighter gray. The animations used in this experiment can be viewed at http://psychology.emory.edu/cognition/wolff/animations.html.

***Procedure***. The animations were presented in random order on Windows-based computers using E-Prime (version 2.0) by Psychology Software tools. Participants were told that they would see a series of animations in which cars bumped into or pulled one another. After each animation, participants chose a sentence that best described the occurrence. All of the sentences named the first and last cars in the causal chain and were the same (*A____ C to [from] cross[ing] the line*) except for the verb, which was *caused, allowed* or *prevented*. Another option was *none of the above*. Participants were instructed to choose the sentence that best described what actually occurred in the scene, not what could have occurred. Participants indicated their answers by clicking a radio button next to their choice.

#### Results and discussion

As shown in the lower two sections of Table 6, participants described the two parts of the causal compositions as intended. For the animations depicting the first and second relations of the A/A chain, participants chose the sentences containing *allow* and *allow*, χ^2^(3, *N* = 24) = 30.2, *p* < 0.0001, χ^2^(2, *N* = 24) = 24.56, *p* < 0.0001; for the animations depicting the first and second relations of the A/C chain, participants chose the sentences containing *allow* and *cause*, χ^2^(2, *N* = 24) = 25.04, *p* < 0.0001, χ^2^(2, *N* = 24) = 6.08, *p* < 0.05; for the animations depicting the first and second relations of the A/P chain, participants chose the sentences containing *allow* and *prevent*, χ^2^(1, *N* = 24) = 6.76, *p* < 0.01, χ^2^(1, *N* = 24) = 21.16, *p* < 0.001; for the animations depicting the first and second relations of the C/C chain, participants chose the sentences containing *cause* and *cause*, χ^2^(2, *N* = 24) = 31.75, *p* < 0.0001, χ^2^(1, *N* = 24) = 18.62, *p* < 0.0001; for the animations depicting the first and second relations of the C/P chain, participants chose sentences containing *cause* and *prevent*, χ^2^(1, *N* = 24) = 12.46, *p* < 0.0001, χ^2^(1, *N* = 24) = 20.17, *p* < 0.0001; for the animations depicting the first and second relations of the P/A chain, participants chose sentences containing *prevent* and *allow*, χ^2^(2, *N* = 24) = 12.00, *p* < 0.01, χ^2^(3, *N* = 24) = 15.67, *p* = 0.001; for the animations depicting the first and second relations of the P/C chain, participants chose the sentences containing *prevent* and *cause*, χ^2^(2, *N* = 24) = 27.91, *p* < 0.0001, χ^2^(2, *N* = 24) = 27.0, *p* < 0.001; and for the animations depicting the first and second relations in the P/P chain, participants chose the sentences containing *prevent* and *prevent*, χ^2^(2, *N* = 24) = 23.25, *p* < 0.0001, χ^2^(1, *N* = 24) = 20.167, *p* < 0.001. In the case of the C/A chain, the most frequently chosen sentence for the first relation was the one containing *cause*, as predicted, but participant's choices in this case did not differ from chance, χ^2^(3, *N* = 24) = 3.25, *p* = 0.355. For the animation depicting the second relation of the C/A chain, participants' modal response was *allow*, χ^2^(2, *N* = 24) = 28.88, *p* < 0.0001.

### Experiment 1b

The goal of this experiment was to examine how people form causal compositions from depictions of causal chains. Participants saw animations of various kinds of causal chains. For example, in one case, participants saw an animation of a CAUSE/CAUSE causal chain involving three cars, A, B, and C. After watching an animation, participants choose the expression that best described the relation between the first and last cars in the chain (e.g., A and C). The expressions named two cars joined by the verbs *cause, allow*, or *prevent*, or the option “none of the above.” Participants' predicted choices are shown in the top half of Table 6.

#### Methods

***Participants***. The participants were 25 Emory University undergraduates who took part in the study for course credit. None of the participants in the current study participated in Experiment 1a. All participants were native speakers of English.

***Materials***. Nine 3D animations were made from an animation package called Autodesk 3 3ds Max 8 as described in Experiment 1a.

***Procedure***. The procedure was the same as in Experiment 1a. Participants watched animations and then chose a sentence that best described what they saw. The sentences named the first and last cars in the animation. The sentence choices were the same as in Experiment 1a.

#### Results and discussion

The predictions of the force theory were fully borne out by the results. The top of Table 6 shows the percentage of times people chose each of the four options for each of the nine chains.

As shown at the top of Table 6, for every type of causal chain, people chose the sentence that matched the relation composition predicted by the force theory. Specifically, for the animation depicting the A/A chain, people chose the sentence contain *allow*, χ^2^(3, *N* = 25) = 21.56, *p* < 0.0001; for the A/C chain, they chose the sentence containing *allow*, χ^2^(3, *N* = 25) = 26.04, *p* < 0.0001; for the A/P chain, they chose the sentence containing *prevent*, χ^2^(3, *N* = 25) = 13.88, *p* < 0.01; for the C/A chain, they chose the sentence containing *allow*, χ^2^(3, *N* = 25) = 13.88, *p* < 0.01; for the C/C chain, they chose the sentence containing *cause*, χ^2^(2, *N* = 25) = 24.56, *p* < 0.0001; for the C/P chain, they chose the sentence containing *prevent*, χ^2^(2, *N* = 25) = 11.56, *p* = 0.001; for the P/A chain, they chose the sentence containing *prevent*, χ^2^(2, *N* = 25) = 25.04, *p* < 0.0001; and for the P/C chain, they chose the sentence containing *prevent*, χ^2^(2, *N* = 25) = 33.68, *p* < 0.0001. For the P/P chain, participants used both *cause* or *allow* verbs, just as predicted by the force theory. Since the percentages for the two choices were the same and participants did not choose any of the other options, chi-square could not be computed.

The results support the assumption of the force theory that the representations specified in the theory are iconic since they could be entered into a physics simulator to produce events that were classified by people as predicted by the theory. In contrast to the predictions made by the mental model theory, people did not indicate that the double preventions should lead to PREVENT relations. According to the force theory and the causal model theory, double preventions can lead to either CAUSE or ALLOW responses, which is exactly what was observed. The results from Experiments 1a,b imply that the simulation of causal chains may be based on mental units that are also capable of supporting a particular kind of causal reasoning in the form of relation composition.

## Abstract causation and forces

The results from Experiments 1 demonstrate how the process of composing causal relations might be based on representations of physical forces, but also suggest how the process of composing causal relations might occur in the case of abstract causal relations, such as *T*ax *cuts cause economic growth* and C*ompetition prevents inflation*. Evidence from cognitive psychology, cognitive linguistics, and cognitive neuroscience has converged on the idea that people may recruit representations from the physical domain to understand the abstract, a proposal that has sometimes been called the *redeployment hypothesis* (Boroditsky, 2001; Gentner et al., 2002; Casasanto and Boroditsky, 2008; DeWall et al., 2010; Chafee and Crowe, 2013; Parkinson et al., 2014). In the case of the force theory, extending it from physical forces to abstract forces (e.g., social forces) requires no change to the underlying processing mechanisms. For example, if told that *Stress causes forgetfulness* and *Forgetfulness causes confusion*, people may represent the component causal relations by imagining CAUSE configurations of forces like the one shown in Figure 2. The forces in this case, would not be physical forces, but instead abstract influences. The magnitude of the vectors would not be precise, but they would not need to be because the force theory already assumes that people do not have access to exact magnitudes of the forces (Wolff, 2007); what they do have is knowledge of the relative magnitudes and directions of the forces. People may represent abstract versions of ALLOW and PREVENT by generating ALLOW and PREVENT configurations of forces. Once abstract relations are represented in terms of configurations of forces, the processes used for composing configurations of forces in the physical world can be recruited for composing configurations of forces in the non-physical world.

Data already exists on the kinds of conclusions people draw when they compose abstract causal relations. In Experiment 4 of Goldvarg and Johnson-Laird (2001), participants were presented with 16 different types of causal chains and were asked to write out a conclusion. To minimize the effect of prior knowledge, the relations in the chains involved abstract psychological terms such as *compliance, anxiety*, and *depression*. Such terms are vague enough that when they are placed in a causal chain, the entire chain sounds plausible. For example, one of the sequences read as follows:

Obedience allows motivation to increase.
Increased motivation causes eccentricity.
What, if anything, follows?

The specific 16 chains are listed in Table 7, along with the relation compositions predicted by the mental model theory, the causal model theory, and force theory. Table 6 also shows the most frequent relation composition chosen by participants in Goldvarg and Johnson-Laird's (2001) Experiment 4.

**Table 7:** **Predicted and observed compositions from Goldvarg and Johnson-Laird (2001) for 16 types of causal chains**.

**Chain**	**C/C**	**C/A**	**C/P**	**C/¬C**	**A/C**	**A/A**	**A/P**	**A/¬C**
Mental model theory	C	A	P	P	A	A	A¬	A¬
Causal model theory	C	A	P	P	A	A	^*^P	^*^P
Force theory	C	A (76%) C (24%)	P	P	A (76%) C (24%)	A	^*^P	^*^P
**(Goldvarg and Johnson-Laird, 2001), Experiment 4 results**	**C (100%)**	**A (95%)**	**P (100%)**	**P (45%)**	**A (90%)**	**A (95%)**	**A¬ (70%)**	**A¬ (60%)**
**Chain**	**P/C**	**P/A**	**P/P**	**P/¬C**	**¬C/C**	**¬C/A**	**¬C/P**	**¬C/¬C**
Mental model theory	P	P	P	C	¬C	¬A	¬P	¬P
Causal model theory	P	P	^*^A (50%) C (50%)	C (50%) A (50%)	¬C	¬A	¬P	¬P
Force theory	P	P	^*^A (62%) C (38%)	C (49%) A (22%)	¬C (50%) ¬A (43%)	¬A (90%) ¬C (9%)	¬P (51%)	¬P (20%)
**(Goldvarg and Johnson-Laird, 2001) Experiment 4 results**	**P (95%)**	**P (100%)**	**P (75%)**	**C (85%)**	**¬C (100%)**	**¬A (100%)**	**¬P (100%)**	**¬P (75%)**

C, “Cause”; A, “Allow”; P, “Prevent”; ¬C, e.g., “Not A cause B”; C¬, e.g., “A cause not C”;

* *missed prediction, Responses in bold, conclusions that were predicted by one of the theories*.

The chains examined in Goldvarg and Johnson-Laird's (2001) Experiment 4 included the nine causal chains examined in Experiment 1a, plus seven others. All told, the mental model theory predicted the modal conclusion produced by the participants for all 16 causal chains. The causal model theory and the force theory also did reasonably well, each accounting for the modal conclusion for 13 of the 16 compositions. These results provide some initial evidence that the predictions of the force theory extend beyond the just the physical domain.

For several chains, the predictions of the force theory, as well as of the causal model theory, diverged from what was observed. One such chain was A/P. Whereas the mental model predicted an ALLOW¬ (i.e., A allows ¬B) conclusion, both the force theory and the causal model theory predicted PREVENT, which was also the result observed in Experiment 1a. Another chain for which the results differed was P/P. As discussed earlier, a sequence of PREVENT relations constitutes double prevention, which, according to many researchers and the results from Experiment 1a, should lead to either ALLOW or CAUSE compositions (Foot, 1967; McMahan, 1993; McGrath, 2003). One reason why participants may have chosen PREVENT over either CAUSE or ALLOW in Goldvarg and Johnson-Laird (2001) is because of the “atmosphere” of the chain (see Bucciarelli and Johnson-Laird, 1999). In particular, participants may have chosen a PREVENT conclusion because the relations in the premises were PREVENT relations, not necessarily because the chain of two PREVENT relations led to a PREVENT composition. The third conclusion missed by both the causal model theory and the force theory was A/¬C (i.e., A allows B, ¬B causes C): the mental model theory predicted the observed modal response of ALLOW¬ (i.e., A allows ¬B), whereas the causal model theory and the force theory predicted the conclusion would be PREVENT. To better understand the nature of these differences, we re-ran Goldvarg and Johnson-Laird's (2001) Experiment 4.

### Experiment 2

The ability of three theories of causal composition to address abstract causation was examined in this experiment. The materials used in this experiment were those used in Goldvarg and Johnson-Laird's (2001) Experiment 4. In particular, all of the materials used in this experiment were based on psychological terms that were highly familiar to the participants but also vague enough that they could be used in different causal statements with different relations and still sound plausible^5^. For instance, as an example of the causal chain P/A, participants read *Learning prevents anxiety, Anxiety allows fixation*. Also as in Goldvarg and Johnson-Laird (2001), negations were encoded with the word *no*. For instance, one example of ¬C read *No learning causes anxiety*. As described below, our analyses include an assessment of the modal response and measures that are sensitive to the distribution of responses (*t*-tests computed over correlations). Both of these measures will be important in our assessment: the modal response equates the theories so that they (broadly speaking) motivate only one (dominant) conclusion, whereas the correlation analysis allows us to assess the more fine-grained predictions of each theory.

In generating the predictions for the three theories in this experiment and in the experiments to follow, we adopted Sloman et al.'s *matching assumption*, which can be viewed as a type of atmosphere effect (see Bucciarelli and Johnson-Laird, 1999), except that the effect does not necessarily lead to an incorrect response. According to Sloman et al. (2009), people are biased to generate causal compositions in which the arguments in the conclusion match those in the premises. Thus, if the first premise is given in terms of an absence, people will prefer to describe the conclusion in terms of an absence. The matching assumption turned out to be a good rule of thumb. However, there are cases for which the matching assumption should probably be relaxed since it occasionally lowers the fit to the data for all of the theories, suggesting the existence of other biases; for example, there may be a bias for conclusions without negations over those with negations.

#### Methods

***Participants***. The participants were 20 Emory University undergraduates who participated for course credit. None of the participants in the current study participated in any of the other experiments described in this paper. All participants were native speakers of English.

***Materials***. The materials were based on two examples each of the 16 causal chains shown in Tables 6, 7. For example, the chain C/C was instantiated by the pairs of sentences *Obedience causes motivation, Motivation causes eccentricity* and *Attention causes rumination, Rumination causes paranoia*. As demonstrated in these examples, the premises were based on psychological terms such as *obedience, motivation*, and *paranoia*. A complete list of the materials is provided in Table A3 in the Appendix.

***Procedure***. The experiment was run on Windows-based computers using Presentation (version 12.2) by Neurobehavioral Systems. Participants were told that the experiment concerned how people reason on the basis of causal knowledge. They were told that they would be presented with reasoning problems that consisted of two statements, such as *Smoking causes cancer, Cancer causes health problems*, which they should assume to be true. Each pair of premises was followed by the question “What, if anything, follows?” For each pair of premises, participants were instructed to type in a response. Participants were asked to think about the premises carefully before drawing a conclusion, and they were allowed to spend as much time as they needed on each problem. Pairs of premises were presented one at a time in a different random order for each participant. The authors coded the participants' responses as to the type of conclusion generated.

***Design***. Participants were randomly assigned one of two lists. Each list contained one half of the materials, which included one example each of the 16 possible compositions.

#### Results and discussion

The results provided support for all three models of causal composition. The predictions of the three models and the percentage of times these predictions were observed are shown in Table 8,^6^. A comparison between these results and those of Goldvarg and Johnson-Laird (2001), reported in Table 7, shows a relatively high level of agreement between the studies. Two cases where the conclusions differed were for the chains A/P and A/¬C. In both cases, the participants in our study strongly preferred PREVENT conclusions, as opposed to ALLOW¬ conclusions in Goldvarg and Johnson-Laird (2001). Another case where the conclusions in the two studies differed was for the chain ¬C/A. In our study, participants' most frequent conclusion was ¬CAUSE (i.e., ¬A causes C), whereas in Goldvarg and Johnson-Laird (2001), it was ¬ALLOW (i.e., ¬A allows C). However, as shown in Table 8, the percentage of ¬CAUSE, 50%, was only slightly higher than that for ¬ALLOW, 40%, so the results observed here do not differ radically from those observed in Goldvarg and Johnson-Laird (2001).

**Table 8:** **Experiment 2 results and predictions by composition and theory**.

**Composition**	**C/C**	**C/A**	**C/P**	**C/¬C**	**A/C**	**A/A**	**A/P**	**A/¬C**
Mental model theory	C	A	P	P	A	A	^*^A¬	^*^A¬
Causal model theory	C	A	P	P	A	A	P	P
Force theory	C	A (76%) C (24%)	P	P	A (76%) C (24%)	A	P	P
**Observed responses**	**C (85%)**	**A (95)**	**P (100%)**	**P (95%)**	**A (65%)**	**A (95%)**	**P (80%)**	**P (70%)**
		**C (5%)**			**C (35%)**		**A¬ (0%)**	**A¬ (0%)**
**Composition**	**P/C**	**P/A**	**P/P**	**P/¬C**	**¬C/C**	**¬C/A**	**¬C/P**	**¬C/¬C**
Mental model theory	P	P	P	C	¬C	^*^¬A	¬P	¬P
Causal model theory	P	P	^*^A (50%) C (50%)	C (50%) A (50%)	¬C	^*^¬A	¬P	¬P
Force theory	P	P	^*^A (62%) C (38%)	C (49%) A (22%)	¬C (50%) ¬A (43%)	^*^¬A (90%) ¬C (9%)	¬P (51%)	¬P (20%)
**Observed responses**	**P (75%)**	**P (80%)**	**P (65%)**	**C (60%)**	**¬C (90%)**	**¬C (50%)**	**¬P (90%)**	**¬P (45%)**
			**A (20%)**	**A (15%)**	**¬A (0%)**	**¬A (40%)**		**C (15%)**
			**C (15%)**	P (15%)				

C, “Cause”; A, “Allow,” P, “Prevent”; ¬C, e.g., “Not A cause B”; C¬ = e.g., “A cause not C”;

* *missed prediction, Observed responses in bold, conclusions that were predicted by one of the theories*.

The mental model theory correctly predicted 13 of the 16 compositions, a result that was significantly greater than chance by a binomial test, *p* < 0.05. The causal model theory and the force theory correctly predicted 14 of the 16 compositions, a result that also differed from chance by a binomial test, *p* < 0.01.

It needs to be acknowledged that for two of the relation compositions, ¬C/P and ¬C/¬C, the force theory predicted the modal response, but at a different level of acceptability than what was observed. For example, for ¬C/P, the force theory predicted that people would produce ¬P at a level of 51%; ¬P was the dominant response, but at a level of 90%. This difference in level of acceptability predicted by the theory and actual responding need not be interpreted as a problem for the force theory. It is quite possible that in cases like ¬C/P, people were not happy with their conclusion, but they went with it anyway because they could not think of another possible response and they did not want to leave the question unanswered.

One prediction that was missed by both the force theory and the causal model was that associated with the chain P/P. In both Goldvarg and Johnson-Laird and in this experiment, the modal response was PREVENT, whereas in Experiment 1a, based on visual materials, the model responses were ALLOW and CAUSE. As already discussed, the force and causal model theories predict ALLOW and CAUSE conclusions. As mentioned earlier, one reason why people may have reached a PREVENT conclusion to chains containing two PREVENTs is because of an atmosphere effect: with two PREVENT relations in the premises, participants may have been biased to report PREVENT conclusions. The potential impact of atmosphere might be especially salient when people do not fully understand the nature of the PREVENT relations in the premises, which is exactly what might be expected given the nature of the materials used in this experiment. As discussed earlier, the materials used vague psychological terms that could be combined in different ways while still sounding plausible. These materials were used to minimize the influence of prior knowledge, but because they were not based on real causal relations, participants may have done less well in instantiating the underlying representations, which may have partially compromised the composition process, making it more vulnerable to atmosphere effects. Our speculation is supported by recent findings that people are more likely to generate CAUSE and ALLOW conclusions to double preventions when the conclusions are supported by prior knowledge (Chaigneau and Barbey, 2008) than when they are not supported by prior knowledge. This finding is consistent with the well-known phenomenon that performance on reasoning problems often improves when participants are given “concrete” or “realistic” content in the task (Wason and Shapiro, 1971; Johnson-Laird et al., 1972; Almor and Sloman, 2000; see Eysenck and Keane, 2000 for a review). The potential benefits of computing causal compositions from known causal relations were examined in the next experiment.

### Experiment 3

The purpose of Experiment 3 was to once again examine the ability of the three models to predict the conclusions that followed from the composition of relatively abstract causal relations. As in Experiment 2, participants drew conclusions from a set of two-premise problems. However, in this experiment, the premises were based on actual causal relationships found in text that could be accessed via the internet. By using such materials, we could investigate the ability of the models to account for complex real world causal relations. Some of the causal relations described physical processes, but the majority of the causal relations were quite abstract (e.g., *Economic freedom causes wealth*). A second difference between the current experiment and Experiment 2 is that in the current experiment the number of chain types was doubled. One final difference between the current experiment and Experiment 2 was in the use of a different dependent measure. Instead of having people type out responses, participants selected from a list of possible responses, such as CAUSE, ALLOW, PREVENT, ¬A_CAUSE, etc. One of the advantages to using a multiple-choice dependent measure is that it highlighted for the participants the full range of possible conclusions that could be derived. A second advantage was that it potentially encouraged participants to spend more time thinking about the conclusions than typing out a response. Minimizing the time requirements of the experiment was important since there were 6 times as many trials in the current experiment than in Experiment 2.

#### Methods

***Participants***. The participants were 40 Emory University undergraduates who took part in the study for course credit. Participants in the current study did not participate in any of the other experiments described in this paper. All participants were native speakers of English.

***Materials***. For each of the 32 causal compositions listed in Table 8, we found six real-world examples for a total of 192 causal chains (see Table A3 in the Appendix). All of the premises used in these chains were found on the internet using the Google and Yahoo search engines^7^.

***Procedure***. Participants were run on windows-based computers in sound-attenuating carrels using E-Prime (version 2.0) by Psychology Software tools. Participants were told that the experiment concerned how people reason on the basis of causal knowledge. They were told that they would be presented with reasoning problems that consisted of two statements such as *Smoking causes cancer, Cancer causes health problems*. Each pair of premises was followed by the question “What, if anything, follows?” For each pair of premises, participants chose from a list of ten possible responses including nine conclusions (CAUSE, ALLOW, PREVENT, ¬A_CAUSE, ¬A_ALLOW, ¬A_PREVENT, CAUSE¬, ALLOW¬, PREVENT¬) and “none of the above.” The list of possible responses was changed to fit the content of each particular composition. For example, for the premises *Sanding wood causes dust, Dust prevents good adhesion*, the possible conclusions included *Sanding causes good adhesion, Sanding allows good adhesion, Sanding prevents good adhesion, Lack of sanding causes good adhesion, Lack of sanding allows good adhesion*, and so on. Participants were told to assume that the premises were true and that they should select the conclusion that best followed from the premises. Pairs of premises were presented, one at a time, in a different random order for each participant.

***Design***. Participants were randomly assigned to one of two list versions. In each, participants made judgments on one half of the materials, which included three examples each of the 32 possible causal chains, for a total of 96 chains.

#### Results and discussion

The results provided stronger support for the force theory and the causal model theories over the mental models theory. The use of real-world materials led to an improvement in the already high fit to the data provided by the force theory and causal model theory. Table 9 lists (in bold) the percentage of times that people chose one of the types of conclusions predicted by at least one of the theories. The mental model theory correctly predicted the most frequent response on 26 of the 32 possible compositions, a result that was significantly greater than chance by a binomial test, *p* < 0.001. The force theory and the causal model theory correctly predicted 30 of the 32 possible compositions, which was also significant by a binomial test, *p* < 0.000001. The Friedman test indicated that the number of correct predictions made by the force theory and the causal model theory was greater than the number of correct predictions made by the mental model theory, χ^2^_(2)_ = 8.00, *p* = 0.018.

**Table 9:** **Experiment 3 results and predictions by composition and theory**.

	**C/C**	**C/A**	**C/P**	**C/¬C**	**A/C**	**A/A**	**A/P**	**A/¬C**
Mental model theory	C	A	P	P	A	A	^*^A¬	^*^A¬
Causal model theory	C	A	P	P	A	A	P	P
Force theory	C	A (76%) C (24%)	P (100%)	P (59%)	A (76%) C (24%)	A	P (22%)	P (5%)
**Observed responses**	**C (78%)**	**A (69%)**	**P (77%)**	**P (62%)**	**A (66%)**	**A (89%)**	**P (69%)**	**P (71%)**
		**C (15%)**			**C (23%)**		**A¬ (10%)**	**A¬ (2%)**
	**P/C**	**P/A**	**P/P**	**P/¬C**	**¬C/C**	**¬C/A**	**¬C/P**	**¬C/¬C**
Mental model theory	P	P	^*^P	C	¬C	¬A	¬P	¬P
Causal model theory	P	P	A (50%) C (50%)	C (50%) A (50%)	¬C	¬A	¬P	¬P
Force theory	P (37%)	P (30%)	A (62%) C (38%)	C (49%) A (22%)	¬C (50%) ¬A (43%)	¬A (90%) ¬C (9%)	¬P (51%)	¬P (20%)
**Observed responses**	**P (93%)**	**P (82%)**	**A (39%)**	**C (58%)**	**¬C (46%)**	**¬A (28%)**	**¬P (38%)**	C (23%)
			**P (30%)**	**A (18%)**	P (26%)	**¬C (13%)**		**¬P (20%)**
			**C (9%)**		**¬A (20%)**			
	**C¬/C**	**C¬/A**	**C¬/P**	**C¬/¬C**	**A¬/C**	**A¬/A**	**A¬/P**	**A¬/¬C**
Mental model theory	P	P	^*^P	C	A¬	A¬	A	A
Causal model theory	P	P	C (50%) A (50%)	C	P	P	A	A
Force theory	P (59%)	P (17%)	C (61%) A (39%)	C (54%) A (13%)	P (5%)	P (69%)	A (95%) C (5%)	A (62%) C (35%)
**Observed responses**	**P (65%)**	**P (73%)**	**C (60%)**	**C (82%)**	**P (61%)**	**P (68%)**	**A (50%)**	**A (60%)**
			**A (28%)**	**A (7%)**	**A¬ (4%)**	**A¬ (8%)**	**C (18%)**	**C (26%)**
			**P (11%)**					
	**P¬/C**	**P¬/A**	**P¬/P**	**P¬/¬C**	**¬C¬/C**	**¬C¬/A**	**¬C¬/P**	**¬C¬/¬C**
Mental model theory	C	A	P	P	¬P	^*^¬P	^*^¬C	¬C
Causal model theory	C (50%) A (50%)	A	P	P	¬P	^*^¬P	^*^¬C	¬C
Force theory	C (49%) A (22%)	A (92%) C (7%)	P (49%)	P (32%)	¬P (19%)	^*^¬P (47%)	^*^¬A (75%) ¬C (23%)	¬C (47%) ¬A (31%)
**Observed responses**	**C (39%)**	**A (53%)**	**P (65%)**	**P (69%)**	**¬P (34%)**	A (43%)	P (37%)	**¬C (34%)**
	**A (20%)**	**C (8%)**			**A (32%)**	**¬P (29%)**	**¬C (30%)**	P (30%)
					**C (21%)**		**¬A (24%)**	**¬A (3%)**

C, “Cause”; A, “Allow”; P, “Prevent”; ¬C, e.g., “Not A cause B”; C¬, e.g., “A cause not C”;

* *missed prediction; Observed responses in bold, conclusions that were predicted by one of the theories*.

The two chains that were missed by the force theory, ¬C¬/A and ¬C¬/P, were also missed by the causal model theory and the mental model theory. The reason why the theories failed to correctly predict these two compositions can be explained by our adoption of the matching processing assumption. As discussed earlier, we assumed that people's conclusions would be expressed in a manner similar to the way they were expressed in the premises. For example, if the first premise was given in terms of an absence, we assumed that people would express the conclusion in terms of an absence. In contrast to this assumption, the modal response to ¬C¬/A was ALLOW, rather than ¬PREVENT, as predicted by all three theories. As it turns out, in all three theories, ¬PREVENT compositions can also be expressed as ALLOW relations^8^. In the case of ¬C¬/P, the modal response was PREVENT, instead of ¬ALLOW as predicted by the force theory, or ¬CAUSE as predicted by the causal model and model theories. As it turns out, the force theory predicts that ¬ALLOW implies PREVENT 100% of the time, and the causal model theory predicts that ¬CAUSE can be re-described as PREVENT. In other words, the mismatch between the predictions of the theories and participants' responses was due to our assumption that participants would describe the conclusions in a manner that matched the premises. If we allow paraphrases of the predicted conclusions, the force theory, and perhaps the causal model theory, are able to predict the responses to all 32 chains.

One particularly important finding in this experiment was with respect to P/P chains. Consistent with Experiment 1a, the most frequently derived conclusion for these chains was ALLOW, not PREVENT. As noted earlier, it has been argued that double preventions should lead to either ALLOW or CAUSE conclusions (Foot, 1967; McMahan, 1993; McGrath, 2003). The current findings, based on real-world causal relations, provide support for this claim.

The results from this experiment suggest that an approach to relation composition based on iconic codes is as good as the causal model theory and better than the mental model theory in being about to predict abstract causal compositions. The force theory's ability to account for causal reasoning was strengthened when the underlying causal relations were based on causal relations with real world correlates, as used in Experiment 2.

In experiments reported so far, the relation compositions involved only two relations. However, all of the theories allow for relation compositions involving three or more relations. In the case of the mental model theory, the composition of C/A/C might begin with the derivation of a conclusion between the first two relations, C and A (leading to an ALLOW conclusion), which could then be composed with the next relation, CAUSE, to yield the overall conclusion ALLOW. As discussed in Sloman et al. (2009), in the causal model theory, the process of substitution can be extended to three-premise compositions and beyond. Finally, in the case of the force theory, adding additional relations simply involves adding together more patient vectors in the conclusion. In the next experiment, we examined the ability of the three models to account for relation compositions involving 3 relations.

### Experiment 4

As in Experiment 4, the materials were based on real-world causal statements obtained from the internet. However, in this experiment, the chains were based on three relations instead of two.

#### Methods

***Participants***. The participants were 24 Emory University undergraduates who took part in the study for course credit. Participants in the current study did not participate in any of the other experiments described in this paper. All participants were native speakers of English.

***Materials***. The materials were based on the 25 causal chains listed in Table 10. We found four real-world examples for each of these Compositions for a total of 100 causal chains (see Table A4 in Appendix A in the Supplementary Materials). As in Experiment 3, all of the premises used in these compositions were found on the internet using the Google and Yahoo search engines.

**Table 10:** **Experiment 4 results and predictions by causal chain and theory**.

	**A/C/C**	**C/A/C**	**C/C/A**	**A/C¬/C¬**	**C¬/A/C¬**	**C¬/C¬/A**	**C/C/C**
Mental model theory	A	A	A	A	^*^C¬	^*^P	C
Causal model theory	A	A	A	A	^*^¬P	A	C
Force theory	A (62%) C (38%)	A (65%) C (35%)	A (69%) C (31%)	A (95%) C (5%)	A (52%) C (48%)	A (93%) C (7%)	C
**Observed responses**	**A (54%)**	**A (58%)**	**A (71%)**	**A (42%)**	**A (25%)**	**A (63%)**	**C (77%)**
	**C (38%)**	**C (27%)**	**C (23%)**	**C (19%)**	**C (21%)**	**C (17%)**	
					**¬P (15%)**	**P (2%)**	
					**C¬ (8%)**		
	**C/C¬/C¬**	**C¬/C/C¬**	**C¬/C¬/C**	**C¬/C/C**	**C/C¬/C**	**C/C/C¬**	
Mental model theory	^*^P	^*^P	^*^P	P	P	C¬	
Causal model theory	C	^*^C	C	P	P	C¬	
Force theory	C (61%) A (39%)	^*^C (60%) A (40%)	C (65%) A (10%)	P	P	C¬	
**Observed responses**	**C (33%)**	**C (18%)**	**C (42%)**	**P (46%)**	**P (42%)**	**C¬ (60%)**	
	**A (31%)**	**A (13%)**	**A (23%)**	C¬ (29%)	C¬ (29%)	**P (25%)**	
	P¬ (8)	P¬ (27)	**P (13%)**				
	**C/P/P**	**P/C/P**	**P/P/C**	**P/C/C**	**C/P/C**	**C/C/P**	
Mental model theory	P	P	P	P	P	P	
Causal model theory	C (50) A (50)	C (50) A (50)	C (50) A (50)	P	P	P	
Force theory	C (61%) A (39%)	A (64) C (37)	C (49) A (22)	P	P	P	
**Observed responses**	**C (35)**	**A (38)**	**C (42)**	**P (83)**	**P (56)**	**P (73)**	
	**A (25)**	**C (4)**	**A (33)**				
	**P (25)**	**P (6)**	**P (4)**				
		**Pn (21)**					
	**A/P/P**	**P/A/P**	**P/P/A**	**C/P/¬C**	**P/C/¬C**	**C/¬C/P**	
Mental model theory	P	P	P	C	C	A	
Causal model theory	A	A	A	C (50) A (50)	C (50) A (50)	C (50) A (50)	
Force theory	A (94) C (5)	A (52%) C (48%)	A (92) C (7)	C (54) A (9)	C (60) A (22)	^*^C (60) A (40)	
	**A (63)**	**A (42)**	**A (60)**	**C (60)**	**C (44)**	**A (42)**	
	**C (0)**	**C (21)**	**C (0)**	**A (19)**	**A (21)**	**C (19)**	
	**P (8)**	**P (13)**	**P (6)**	P (2)	P (4)	P (23)	

C, “Cause,” A, “Allow”; P, “Prevent”; ¬C, e.g., “Not A cause B”; C¬ = e.g., “A cause not C”;

* *missed prediction; Observed responses in bold, conclusions that were predicted by one of the theories*.

***Procedure and Design***. The procedure was essentially the same as in Experiment 3, except that participants were presented with three connected premises instead of two. For example, people saw statements like *Factories cause pollution. Pollution causes global warming. Global warming prevents snow fall*. Below each triplet were ten possible responses including nine conclusions (e.g., *Factories cause snow fall, Factories allow snow fall, Factories prevent snow fall, Lack of factories cause snow fall, Lack of factories allow snow fall, Lack of factories prevent snow fall, Factories cause lack of snow fall, Factories allow lack of snow fall, Factories prevent lack of snow fall*) and *None of the above*. Participants were told to assume that the premises were true and that they should select the conclusion that best followed from the premises. Pairs of premises were presented, one at a time, in a different random order for each participant. The list of causal chains was divided in half, with each list containing two examples of each type of causal chain. Half of the participants saw one list and the remaining participants saw the other list.

#### Results and discussion

The results provided further evidence that people may reason about causal chains using iconic codes, even when the component causal relations are complex and abstract. The results are shown in Table 10, which lists (in bold) the percentage of times that people chose one of the types of conclusions predicted by at least one of the theories.

The mental model theory correctly predicted the most frequent response in 14 of the 25 possible compositions, a result that did not differ from chance by a binomial test, *p* = 0.69. The force theory and the causal model theory correctly predicted 23 of the 25 possible compositions, a result that differed from chance by a binomial test, *p* < 0.001. The Friedman test indicated that the number of correct predictions made by the force theory and the causal model theory was greater than the number of correct predictions made by the mental model theory, χ^2^_(2)_ = 18.00, *p* < 0.001.

One chain that was missed by all three theories was C¬/C/C¬. The mental model theory predicts that the modal response should be PREVENT while the causal model and the force theory predict that it should be CAUSE. The actual modal response was PREVENT¬. As it turns out, both the causal model and force theory predict that PREVENT¬ is related to CAUSE (because PREVENT¬ is a way of expressing a double prevention), so PREVENT¬ conclusions were not at all inconsistent with these theories.

One chain that both the mental model and the causal model theory missed was C¬/A/C¬. The mental model theory predicted CAUSE¬ and the causal model theory predicted ¬PREVENT, whereas the actual modal response was ALLOW. As described in Sloman et al. (2009), C¬/A/C¬ implies D:= ~(~A and X), which cannot be re-described as D:= A and X, and hence the conclusion ¬PREVENT cannot be re-expressed as ALLOW.

The second chain missed by the force theory was C/¬C/P. The force theory predicted the conclusions CAUSE (60%) and ALLOW (40%) while the observed conclusions were ALLOW (42%) and CAUSE (19%). The causal model theory also predicted CAUSE and ALLOW, but not which conclusion should occur more frequently. As it turns out, of the six cases for which the causal model theory predicted both CAUSE and ALLOW, the C/¬C/P chain was the only chain for which the force theory did predict the most frequent conclusion. Indeed, given that the causal model theory does not specify which conclusion should be more frequent in chains where both CAUSE and ALLOW conclusions are predicted, it could be argued that the causal model theory only correctly predicted 18 of the 25 modal responses, in contrast to the force theory's 23 out of 25. In sum, the results indicate that the force theory provides at least as good account of people's causal compositions as the causal model theory, and a better account than the mental model theory.

## General discussion

In this paper we investigated three accounts of causal composition. One of the main questions addressed in this research was whether generative causal reasoning could be accomplished in terms of iconic mental codes, or whether such reasoning necessarily involved the use of abstract mental codes. The results from Experiment 1 provide strong support for the force theory over the causal model and mental model theories. All of the theories did a good job of predicting how the causal chains would be composed, but only the force theory was able to be able to predict how such chains might be instantiated in the physical world. This is important because the ability to predict how the chains might be instantiated in the world can be reversed to explain how such events might be perceived. In the other theories, additional machinery must be formulated to explain how the representations used in these theories are related to perceptual experience. The remaining experiments examined whether reasoning on the basis of iconic representations could account for the way people compose abstract causal relations. The results in Experiment 2 showed that reasoning on the basis of iconic representations could account for relation composition just as well as reasoning on the basis of abstract representations. The results from Experiments 3, 4 showed that when the relations being composed were associated with actual causal relations, an account based on iconic mental codes was able to explain the data as well as or better than accounts based on abstract mental codes.

## Significance of causal composition

Theories of relation composition allows us to address a number of theoretical challenges in the causal representation and reasoning literatures. For one, theories of relation composition help explain why causal relations sometimes require spatial and temporal contiguity, while other times they do not (Einhorn and Hogarth, 1986). The importance of spatial and temporal contiguity has been repeatedly observed in standard “billiard ball” type events in which one object hits and launches another into motion (Michotte, 1946/1963; Leslie, 1984; for a review, see Scholl and Tremoulet, 2000; Hubbard, 2013a,b). But clearly, in other scenarios, spatial-temporal contiguity is not needed. To borrow an example from Davidson (2001, p. 177), putting poison in the water tank of a spaceship while it is still on earth constitutes murder even if the astronauts do not drink the water and die until they reach Mars. Relation composition offers a way to address this non-contiguity: when a causal relation consists of a single relation, spatial-temporal contiguity is necessary, but when a causal relation is derived from the composition of causal relations, spatial-temporal contiguity is not necessary (Wolff, 2007).

Another phenomenon that theories of relation composition help us to explain is the hierarchical structure of causal knowledge. The process of relation composition results in an over-arching causal relation. This summary relation can then serve as a component in another causal chain, which, in turn, might be chunked into an even more abstract causal relation (Keil, 2006; Walsh and Sloman, 2011).

Yet another reason why causal relation composition may be essential to our understanding of causal knowledge is that it may support a special kind of causal learning. Consider, for example, how people might learn the causal relation *overgrazing causes desertification*. At first, overgrazing causes the lack of vegetation. Once the vegetation is removed, the dry unprotected soil is blown away by the wind or washed away by water, leaving the lower, infertile soil that prevents the re-establishment of plants. Eventually, the dry rocky soil forms dunes (Wilson, 2002). The causal relationship between overgrazing and desertification was probably not learned by directly observing a statistical dependency between overgrazing and desertification; the time periods involved are too long. Instead, such relations are probably learned though the composition of simple causal relations into more complex causal relations. When people acquire causal relations from the composition of relations, it illustrates *learning by reasoning* (Barbey and Wolff, 2007). Learning by reasoning allows for the online construction of causal structures from various snippets of causal knowledge (di Sessa, 1993; Barsalou, 1999; Keil, 2006).

## Conclusion

Much of the recent research on causal cognition has been dominated by theories of causation that assume non-iconic representations. The emphasis is quite understandable since such theories have been able to address in a rigorous fashion some of the most interesting aspects of causal cognition, in particular, the problems of how people put causal relationships together into larger structures and then reason over those structures. The force theory represents the first iconic-based theory to address these more demanding problems. The results from this paper show that a process approach is not only able to address these kinds of problems, but may also offer a stronger fit to the data than accounts based on abstract representations, demonstrating how the units of cognition that are generated from perceptual processes may enter directly into higher-order reasoning.

### Conflict of interest statement

The authors declare that the research was conducted in the absence of any commercial or financial relationships that could be construed as a potential conflict of interest.

## Footnotes

^1^When the causal model theory is applied to situations in which the variables are binary and the relations are deterministic, the truth conditions of the equations can be derived from Boolean algebra. Thus, the equation B:= A is consistent with B = true and A = true, B = false and A = false, and is not consistent with B = true and A = false or B = false and A = true. Although the causal model theory reduces to Boolean algebra in this context, Sloman et al. (2009) note that the causal model theory remains relevant. First, it provides the motivation for the proposed structural equations and the fact that causal graphs can be used directly to derive predictions. Second, causal model theory can be applied to a range of arguments including arguments involving uncertainty, different structural relations, intervention, and arguments with arbitrarily-valued variables (see Hitchcock, 2001).

^2^The availability of a construal that highlights just the initial and final elements of causal chain has been referred to in the linguistics literature as *windowing of attention* (Talmy, 2000). The phenomenon was also noted by Michotte (1946/1963) as the *tool effect*: In a three object chain, A appears to launch C while B simply “drops out” as if it were a tool for A (Hubbard, 2013a).

^3^In the following experiments, all references to the concept of ALLOW assume that the underlying forces form a double prevention. We limited the forces underlying double prevention to those leading to an ALLOW conclusion in a qualitative manner, specifically, by only using the patient vector in the second premise of the double prevention. We further assume the resultant of an ALLOW relation that is passed on to the next relation is based on the vector addition of all of the (non-derived) forces in the two PREVENT relations, not just the resultant of the last PREVENT relation in the double prevention. It is in this way that the two prevention relations are treated as a single unit in the causal chain.

^4^When the magnitude of the patient is 0, the configuration of forces is potentially compatible with *A CAUSES¬B* but not *A PREVENTS B*, and it is because of this one difference that the two expressions are not perfectly synonymous.

^5^ We thank Eugenia Goldvarg-Steingold and Phil Johnson-Laird for access to their materials.

^6^The percentages in Tables 8–10 do not always sum to 100% because participants sometimes indicated that no conclusion followed (Experiment 2) or chose “none of the above” (Experiments 3 and 4).

^7^ Finding an example typically began with typing in a causal verb, sometimes with a negation, such as *cause, allow, prevent, lack of causes, absence of causes, causes lack of*, and then looking for results with an *A-cause-verb-B* structure. We sought examples in which the A and B terms consisted of only one or two words. Sometimes it was possible to shorten longer phrases into one or two words. For example, the sentence *Scanning probe microscopes allow observation of insulating organic materials* was re-written as simply *Microscopes allow observation*. In effect, the process of sampling the A and B arguments involved finding the “head” noun of the A and B term phrases, that is, the main noun that is modified by other elements in a noun phrase (Radford, 1988). Once a possible premise was identified, we searched for a second premise that could be linked to it. For example, given the premise *Microscopes allow observation*, we looked for phrases containing a causal verb, either with or without negation, that either ended with *microscopes* or began with *observation*. We used search terms such as *cause microscopes, allows microscopes, observation causes, observation allows*, etc. As with the initial premise, the arguments in the second premise sometimes needed to be reduced to their head nouns.

^8^For ¬C¬/A, the causal model theory implies C:= ~(~A) and X, which is equivalent to ¬PREVENT (Sloman et al., 2009); however the composition can also be re-described as C:= A and X, which maps onto ALLOW.
