# Supplementary material for: Causal reasoning with forces
Source: Front Hum Neurosci. 2015 Jan 21;9:1. doi: 10.3389/fnhum.2015.00001 (PMC4301188; doi:10.3389/fnhum.2015.00001)
Supplement: Supplementary file 1 [file DataSheet1.DOCX]

| Table A1. Magnitude of the forces acting on the cars in Experiment 1. | | | | | | | | | |
| --- | --- | --- | --- | --- | --- | --- | --- | --- | --- |
| **Forces** | **A**/**A** | **A**/**C** | **A**/**P** | **C**/**A** | **C**/**C** | **C**/**P** | **P**/**A** | **P**/**C** | **P**/**P** |
| **A** | 3N, right | 3N, right | 4.5N, right | 4N, right | 5N, right | 4.5N, right | 5N, right | 4N, right | 5N, right |
| **B** | 3N, left | 2.5N, left | 1N, left | 1N, left | 3.5N, left | 4N, left | 4N, left | 3N, left | 4N, left |
| **C** | 2.5N, right | 1N, right | 1N, right | 2.5N, left | 1.5N, left | 2N, left | 3N, right | 2.5N, right | 2.5N, right |
| **D** | 2.5N, left | 0.5N, left | 0.5N, left | 2.5N, right | - | - | 2.5N, left | - | - |
| **E** | 1.5N, right | - | - | - | - | - | - | - | - |

Note. N = newton’s

Table A2. Materials used in Experiment 2.

| **Compositions** | **First premise** | **Second Premise** |
| --- | --- | --- |
| Allow ◦ Allow | Stress allows compliance. | Compliance allows confusion. |
|  | Counseling allows creativity. | Creativity allows support. |
| Allow ◦ Cause | Learning allows anxiety. | Anxiety causes fixation. |
|  | Attention allows rumination. | Rumination causes paranoia. |
| Allow ◦ ¬A_Cause | Addiction allows distress. | No-distress causes sensitivity. |
|  | Attention allows distress. | No distress causes sensitivity. |
| Allow ◦ Prevent | Insomnia allows distrust. | Distrust prevents depression. |
|  | Obedience allows motivation. | Motivation prevents eccentricity. |
| Cause ◦ Allow | Social-reinforcement causes coping. | Coping allows self-efficacy. |
|  | Insomnia causes distrust. | Distrust allows depression. |
| Cause ◦ Cause | Attention causes rumination. | Rumination causes paranoia. |
|  | Perception causes memory. | Memory causes relaxation. |
| Cause ◦ ¬A_Cause | Perception causes memory. | No-memory causes relaxation. |
|  | Learning causes anxiety. | No anxiety causes fixation. |
| Cause ◦ Prevent | Obedience causes motivation. | Motivation prevents eccentricity. |
|  | Self reinforcement causes coping. | Coping prevents self efficacy. |
| ¬A_Cause ◦ Allow | No insomnia causes distrust. | Distrust allows depression. |
|  | No self reinforcement causes coping. | Coping allows self efficacy. |
| ¬A_Cause ◦ Cause | No perception causes memory. | Memory causes relaxation. |
|  | No attention causes rumination. | Rumination causes paranoia. |
| ¬A_Cause ◦ ¬A_Cause | No addiction causes distress. | No-distress causes sensitivity. |
|  | No obedience causes motivation. | No motivation causes eccentricity. |
| ¬A_Cause ◦ Prevent | No attention causes rumination. | Rumination prevents paranoia. |
|  | No learning causes anxiety. | Anxiety prevents fixation. |
| Prevent ◦ Allow | Learning prevents anxiety. | Anxiety allows fixation. |
|  | Perception prevents memory. | Memory allows relaxation. |
| Prevent ◦ Cause | Obedience prevents motivation. | Motivation causes eccentricity. |
|  | Insomnia prevents distrust. | Distrust causes depression. |
| Prevent ◦ ¬A_Cause | Social-reinforcement prevents coping. | No coping causes self-efficacy. |
|  | Addiction prevents distress. | No distress causes sensitivity. |
| Prevent ◦ Prevent | Stress prevents compliance. | Compliance prevents confusion. |
|  | Insight prevents consistency. | Consistency prevents morality. |

Table A3. Materials used in Experiment 3.

| **Compositions** | **First premise** | **Second Premise** |
| --- | --- | --- |
| Allow ◦ Allow | Licenses allow sales. | Sales allow production. |
|  | Microscopes allow observation. | Observation allows identification. |
|  | Training allows access. | Access allows options. |
|  | Satellites allow voice communication. | Voice communication allows complex commands. |
|  | Security flaws allow identity theft. | Identity theft allows stealing. |
|  | The internet allows information exchange. | Information exchange allows cooperative processes. |
| Allow ◦ Cause | Discrimination allows poverty. | Poverty causes illness. |
|  | Higher revenue allows for tax cuts. | Tax cuts cause economic growth. |
|  | Warm weather allows construction. | Construction causes traffic congestion. |
|  | General public license allows for profit sharing. | Profit sharing causes higher profits. |
|  | Modern societies allow free speech. | Free speech causes harm. |
|  | Pension plans allow for retirement. | Retirement causes happiness. |
| Allow ◦ ¬A_Cause | Grants allow research. | Lack of research causes ignorance. |
|  | Mixed zoning allows storage facilities. | Lack of storage facilities causes food shortages. |
|  | Reduced resistance allows blood flow. | Lack of blood flow causes heart attacks. |
|  | Compromise allows diplomacy. | Absence of diplomacy causes civil war. |
|  | Multi-family housing allows affordable housing. | Lack of affordable housing causes homelessness. |
|  | Mutual trust allows leadership. | Lack of Leadership causes low morale. |
| Allow ◦ Prevent | Email allows communication. | Communication prevents misunderstanding. |
|  | Privatization allows competition. | Competition prevents inflation. |
|  | Sensors allow heating. | Heating prevents condensation. |
|  | Buses allow travel. | Travel prevents stagnation. |
|  | Statistics allow analysis. | Analysis prevents disaster recurrence. |
|  | Water activities allow exercise. | Exercise prevents arthritis. |
| Allow¬ ◦ Allow | Black holes allow no escape. | Escape allows hope. |
|  | Self-centeredness allows lack of empathy. | Empathy allows understanding. |
|  | Unending workload allows no leisure. | Leisure allows self-actualization. |
|  | Certain fabrics allow no moisture absorption. | Moisture absorption allows swelling. |
|  | Divine mysteries allow no rational analysis. | Rational analysis allows proper assessments. |
|  | Strict control allows no compromise. | Compromise allows resolution. |
| Allow¬ ◦ Cause | Amateur Radio allows no broadcasting. | Broadcasting causes interference. |
|  | Obedience allows the absence of feeling. | Feeling causes attachment. |
|  | Vegan diets allow no animal products. | Animal products cause cholesterol. |
|  | Computer indexation allows lack of artificial limits. | Artificial limits cause surpluses. |
|  | Socialism allows no private property. | Private property causes inequality. |
|  | Technology allows an absence of friction. | Friction causes heat. |
| Allow¬ ◦ ¬A_Cause | Insurance allows lack of experience. | Lack of experience causes mistakes. |
|  | Leadership allows not knowing. | Not knowing causes anxiety. |
|  | System sciences allow absence of central control. | Absence of central control allows less bureaucracy. |
|  | Colleges allow lack of self discipline. | Lack of self-discipline causes mental suffering. |
|  | Regimes allow no freedom. | No freedom causes resentment. |
|  | The internet allows a lack of quality. | Lack of quality causes expenses. |
| Allow¬ ◦ Prevent | Military regimes allow no dissent. | Dissent prevents bad decisions. |
|  | War allows a lack of accountability. | Accountability prevents corruption. |
|  | Warm climates allow a lack of clothing. | Clothing prevents mosquito bites. |
|  | Consensus allows lack of change. | Change prevents problems. |
|  | Corporate philosophy allows a lack of scientific rigor. | Scientific rigor prevents over-generalization. |
|  | Self-government allows no excuses. | Excuses prevent achievement. |
| Cause ◦ Allow | Economic freedom causes wealth. | Wealth allows risk taking. |
|  | Meditation causes relaxation | Relaxation allows healing. |
|  | Marriage causes better health | Better health allows more income. |
|  | Dams cause lakes. | Lakes allow boating. |
|  | Economic development causes democracy. | Democracy allows debate. |
|  | Water causes rot. | Rot allows mold. |
| Cause ◦ Cause | Cell phones cause car accidents. | Car accidents cause delays. |
|  | Factories cause pollution. | Pollution causes global warming. |
|  | Nerve damage causes pain. | Pain causes lost workdays. |
|  | Garlic causes bad breath. | Bad breath causes embarrassment. |
|  | Stress causes forgetfulness. | Forgetfulness causes trouble. |
|  | Tides cause currents. | Currents cause wind. |
| Cause ◦ ¬A_Cause | Cathodes cause light. | Absence of light causes darkness. |
|  | Chemical reactions cause electricity. | Lack of electricity causes civil unrest. |
|  | Self-confidence causes motivation. | Lack of motivation causes underachievement. |
|  | Air pressure causes wind. | Lack of wind causes smoke problems. |
|  | Price increases cause profit. | Lack of profit causes business failure. |
|  | Tobacco advertising causes smoking. | Absence of smoking causes weight gain |
| Cause ◦ Prevent | Graphics transmissions cause internet crashes. | Internet crashes prevent email transmission. |
|  | Merger causes confusion. | Confusion prevents accountability. |
|  | Nervousness causes caution. | Caution prevents suffering. |
|  | Cold winds cause blizzards. | Blizzards prevent travel. |
|  | Poor management causes deforestation. | Deforestation prevents plant life. |
|  | Sanding wood cause dust. | Dust prevents good adhesion. |
| Cause¬ ◦ Allow | Carbon monoxide causes lack of oxygen. | Oxygen allows fire. |
|  | Heroin causes lack of concentration. | Concentration allows deeper exploration. |
|  | Pain causes lack of sleep. | Sleep allows healing. |
|  | Alcohol causes lack of resolve. | Resolve allows persistence. |
|  | Dependency causes lack of confidence. | Confidence allows expression. |
|  | Disconnectivity causes lack of functionality. | Functionality allows advanced searching. |
| Cause¬ ◦ Cause | Famine causes lack of food. | Food causes growth. |
|  | Surgery causes lack of sensation. | Sensation causes alertness. |
|  | Welfare causes lack of income. | Income causes prestige. |
|  | Chemical dependence causes lack of productivity. | Productivity causes profitability. |
|  | Laziness causes lack of work. | Work causes bone damage. |
|  | Leaf loss causes lack of shade. | Shade causes cooling. |
| Cause¬ ◦ ¬A_Cause | Alienation causes lack of support. | Lack of support causes dissatisfaction. |
|  | Hookworm causes lack of iron. | Lack of iron causes tiredness. |
|  | Old age causes lack of imagination. | Lack of imagination causes foolish decisions. |
|  | Broken bones and causes lack of mobility. | Lack of mobility causes stiffness. |
|  | Ignorance causes lack of skepticism. | Lack of skepticism causes harm. |
|  | Isolation causes lack of diversity. | Lack of diversity causes staleness. |
| Cause¬ ◦ Prevent | Chemotherapy causes lack of appetite. | Appetite prevents vitamin deficiencies. |
|  | Greed causes lack of truth. | Truth prevents self-destruction. |
|  | Hot soil causes lack of seed germination. | Seed germination prevents soil crusting. |
|  | Brevity causes lack of precision. | Precision prevents misinterpretation. |
|  | Intelligence failure causes lack of confidence. | Confidence prevents changing course. |
|  | High energy causes lack of attention. | Attention prevents mishaps. |
| ¬A_Cause ◦ Allow | Absence of democracy causes of terrorism. | Terrorism allows atrocities. |
|  | Absence of free radicals causes aging. | Aging allows abnormal skin formation. |
|  | Lack of morals causes criminal behavior. | Criminal behavior allows drug problems. |
|  | Absence of competition causes high profits. | High profits allow capital investment. |
|  | Absence of stress causes muscle relaxation. | Muscle relaxation allows heat dissipation. |
|  | Lack of knowledge causes confusion. | Confusion allows cheating. |
| ¬A_Cause ◦ Cause | Lack of education causes poverty. | Poverty causes social problems. |
|  | Lack of ozone allows harmful radiation. | Harmful radiation causes cancer. |
|  | Lack of zinc causes wilting. | Wilting causes premature deterioration. |
|  | Absence of nicotine causes withdrawal. | Withdrawal causes headaches. |
|  | Lack of fitness causes snoring. | Snoring causes marital discord. |
|  | Lack of light causes depression. | Depression causes loss of appetite. |
| ¬A_Cause ◦ ¬A_Cause | Absence of fathers causes violence. | Absence of violence causes peace. |
|  | Absence of zoning regulations causes flooding | Absence of flooding causes river narrowing. |
|  | Lack of rain causes concern. | Lack of concern causes acceptance. |
|  | Lack of food causes suffering. | Lack of suffering causes great relief. |
|  | Lack of physical activity causes body fat. | Lack of body fat causes coldness. |
|  | Lack of ventilation causes humidity. | Lack of humidity causes brittle hair. |
| ¬A_Cause ◦ Prevent | Lack of food causes metabolism slowdown. | Metabolism slowdown prevents weight loss. |
|  | Absence of gravity causes muscle atrophy. | Muscle atrophy prevents neuromuscular junctions. |
|  | Lack of funding causes delay | Delay prevents timely decisions. |
|  | Lack of focus causes uncertainty. | Uncertainty prevents action. |
|  | Lack of good air causes headaches. | Headaches prevent concentration. |
|  | Lack of rainfall causes drought. | Drought prevents mosquito breeding. |
| ¬A_Cause ¬ ◦ Allow | Lack of honorifics causes lack of harmony. | Harmony allows balance. |
|  | Lack of love causes a lack of life. | Life allows exploration. |
|  | Lack of rainfall causes the lack of vegetation. | Vegetation allows wildlife. |
|  | Absence of knowledge causes a lack of engagement. | Engagements allow benefits. |
|  | Lack of acceptance causes a lack of security. | Security allows protection. |
|  | Lack of motivation causes the lack of innovation. | Innovation allows modernization. |
| ¬A_Cause ¬ ◦ Cause | Lack of learning causes a lack of brain growth. | Brain growth causes intelligence. |
|  | Lack of religion causes a lack of social responsibility. | Social responsibility causes financial success. |
|  | Lack of sun causes lack of blooms. | Blooms allow water discoloration. |
|  | Lack of experience causes a lack of efficiency. | Efficiency causes overall advancement. |
|  | Lack of latent heat causes the lack of cloud development. | Cloud development causes surface cooling. |
|  | Lack of money causes the lack of demand. | Demand causes price increases. |
| ¬A_Cause¬ ◦ ¬A_Cause | Lack of fitness causes lack of attention. | Lack of attention causes inaccuracies. |
|  | Lack of oil causes lack of lubrication. | Lack of lubrication causes excessive wear. |
|  | Lack of physical training causes lack of balance. | Lack of balance causes bruises. |
|  | Lack of funding causes a lack of personnel. | Lack of personnel causes overwork. |
|  | Lack of neural signals causes absence of movement. | Lack of movement causes stiffness. |
|  | Lack of vitamins causes lack of energy. | Lack of energy causes depression. |
| ¬A_Cause¬ ◦ Prevent | Lack of B12 causes lack of oxygen. | Oxygen prevents odors. |
|  | Lack of clarity causes a lack of structure. | Structure prevents turf wars. |
|  | Lack of endorphins also causes a lack of hop | Hope prevents early death. |
|  | Lack of accessibility causes a lack of availability. | Availability prevents productivity loss. |
|  | Lack of goals causes a lack of success. | Success prevents unnecessary expense. |
|  | Lack of thelaws causes absence of regulatory framework. | Regulatory frameworks prevent harm. |
| Prevent ◦ Allow | Altitude prevents cultivation. | Cultivation allows livestock production. |
|  | Civilization prevents evolution. | Evolution allows adaptation |
|  | Strict budgets prevent new technology. | New technology allows monitoring. |
|  | Conflict prevents investment. | Investment allows expansion. |
|  | Standardization prevents innovation. | Innovation allows modernization. |
|  | Tariffs prevent trade. | Trade allows specialization. |
| Prevent ◦ Cause | Nuts prevent blood clots. | Blood clots cause strokes. |
|  | Spark detectors prevent explosions | Explosions cause intense fire. |
|  | Vegetation prevents erosion. | Erosion causes landslides. |
|  | Encryption prevents identify theft. | Identify theft causes misery. |
|  | Milk prevents diabetes. | Diabetes causes foot problems. |
|  | Rubberized coatings prevent slipping. | Slipping causes vibrations. |
| Prevent ◦ ¬A_Cause | Phosphoric acid prevents calcium. | Lack of calcium causes dark spots. |
|  | Psychiatric impairments prevent employment. | Lack of employment causes crime. |
|  | Regulatory policies prevent public transit. | Lack of public transit causes pedestrian injuries. |
|  | Cigarettes prevent proper nutrition. | Lack of nutrition causes hair loss. |
|  | Insulin prevents protein breakdown. | Lack of protein breakdown causes ADHD. |
|  | Politics prevents doubt. | Lack of doubt cause religious intolerance. |
| Prevent ◦ Prevent | Green tea prevents Alzheimer's | Alzheimer's prevents remembering. |
|  | Police prevent crime. | Crime prevents socializing. |
|  | Time management prevents stress. | Stress prevents clear thinking. |
|  | Competition prevents of inflation. | Inflation prevents revenues. |
|  | Customs prevents smuggling. | Smuggling prevents progress. |
|  | Dryness prevents vegetation. | Vegetation prevents erosion. |
| Prevent¬ ◦ Allow | River flow prevents a lack of safe water. | Safe water allows swimming. |
|  | Testing prevents lack of consumer involvement. | Consumer involvement allows decision making. |
|  | Watermarks prevent a lack of acknowledgment. | Acknowledgement allows confirmation. |
|  | Communication prevents a lack of transparency. | Transparency allows public scrutiny. |
|  | Transparent management prevents a lack of accountability. | Accountability allows effective compliance. |
|  | Tree replacement prevents the lack of wood. | Wood allows flexible designs. |
| Prevent¬ ◦ Cause | Beverages prevent a lack of energy. | Energy causes chemical changes. |
|  | Correct positioning prevents a lack of fuel flow. | Fuel flow causes turbulence. |
|  | Water currents prevent lack of oxygen. | Oxygen causes decomposition. |
|  | Education prevents the lack of skilled workers. | Skilled workers cause expanded production. |
|  | Fertilizer prevents the lack of carbon. | Carbon causes global warming. |
|  | Salt prevents the lack of iodine. | Iodine causes stains. |
| Prevent¬ ◦ ¬A_Cause | Clear laws prevent lack of tolerance. | Lack of tolerance causes hate. |
|  | Reservoirs prevent lack of water. | Lack of water causes muscle soreness. |
|  | Rules prevent lack of standardization. | Lack of standardization causes confusion. |
|  | Libraries prevent the lack of information. | Lack of information causes fear. |
|  | Sea salts prevent a lack of magnesium. | Lack of magnesium causes weak muscles. |
|  | Vegetables prevent a lack of nutrients. | Lack of nutrients causes weight loss. |
| Prevent¬ ◦ Prevent | Consistency prevents lack of trust. | Trust prevents uncertainty. |
|  | Laughter prevents a lack of compassion. | Compassion prevents prejudice. |
|  | Proper diet prevents lack of protein. | Protein prevents skin tumors. |
|  | Fertilizer prevents lack of nitrogen. | Nitrogen prevents scorched grass. |
|  | Judges prevent a lack of proper defense. | Proper defense prevents. |
|  | Supplements prevent a lack of vitamin D. | Vitamin D prevents fractures. |

Table A4. Materials used in Experiment 4.

| **Compositions** | **First premise** | **Second Premise** | **Third Premise** |
| --- | --- | --- | --- |
| Allow ◦ Cause ◦ Cause | Higher revenue allows for tax cuts. | Tax cuts cause economic growth. | Economic growth causes inflation. |
|  | Public licenses allow for profit sharing. | Profit sharing causes higher profits. | Higher profits cause more investment. |
|  | Innovation allows adaptation. | Adaptation causes evolution. | Evolution causes specialization. |
|  | Bars allow smoking. | Smoking causes cancer. | Cancer causes depression. |
| Cause ◦ Allow ◦ Cause | Dams cause lakes. | Lakes allow fishing. | Fishing causes animal suffering. |
|  | Laughter causes deep breathing. | Deep breathing allows relaxation. | Relaxation causes sleep. |
|  | Summer causes warm weather. | Warm weather allows construction. | Construction causes traffic congestion. |
|  | Water causes rot. | Rot allows mold. | Mold causes allergies. |
| Cause ◦ Cause ◦ Allow | Investment causes economic development. | Economic development causes democracy. | Democracy allows debate. |
|  | Blizzards cause snow. | Snow cause school closures. | School closures allow snowball fights. |
|  | Tides cause currents. | Currents cause erosion. | Erosion allows plant growth. |
|  | Viruses cause disease. | Disease causes weight loss. | Weight loss allows frailty. |
| Allow ◦Cause¬ ◦ Cause¬ | Passwords allow access. | Access causes lack of delays. | Delays cause lack of momentum. |
|  | Privatization allows competition. | Competition causes lack of inflation. | Inflation causes lack of revenues. |
|  | Many years in the same house allows clutter. | Clutter causes lack of focus. | Focus causes lack of feeling. |
|  | Thin walls allow noise. | Noise causes lack of concentration. | Concentration causes lack of mistakes. |
| Cause¬ ◦ Allow ◦ Cause¬ | Workload causes the absence of parents | Parents allow poor diets. | Poor diets cause lack of energy |
|  | Communism causes lack of religion. | Religion allows spirituality. | Spirituality causes lack of depression. |
|  | Relationships cause the absence of unhappiness. | Unhappiness allows apathy. | Apathy causes lack of awareness |
|  | Disability causes lack of working. | Working allows training. | Training causes lack of mistakes. |
| Cause¬ ◦ Cause¬ ◦ Allow | Ignorance causes lack of doubt. | Doubt causes lack of confidence. | Confidence allows growth. |
|  | Mountain barriers cause lack of precipitation | Precipitation causes lack of adequate friction. | Friction allows stopping. |
|  | Caffeine causes lack of drowsiness. | Drowsiness cause lack of awareness | Awareness allows identification of inner experiences. |
|  | Vaccines cause lack of flu. | Flu causes lack of play. | Play allows exploration. |
| Cause ◦ Cause ◦ Cause | Cell phones cause car accidents. | Car accidents cause delays. | Delays cause frustration. |
|  | Heavy rain causes floods. | Floods cause havoc. | Havoc causes panic. |
|  | Radiation causes nerve damage. | Nerve damage causes pain. | Pain causes lost workdays. |
|  | School causes stress. | Stress causes forgetfulness. | Forgetfulness causes trouble. |
| Cause ◦ Cause¬ ◦ Cause¬ | Kickboxing causes brain damage. | Brain damage causes lack of self-control. | Self-control causes lack of fear. |
|  | Merger causes confusion. | Confusion causes lack of accountability. | Accountability causes lack of arbitrary decisions. |
|  | Nervousness causes caution. | Caution causes lack of suffering. | Suffering causes lack of happiness. |
|  | Stigma causes shame. | Shame causes lack of honesty. | Honesty causes lack of corruption. |
| Cause¬ ◦ Cause ◦ Cause¬ | Encryption causes lack of identity theft. | Identity theft causes misery. | Misery causes lack of smiling. |
|  | Isolation causes lack of marriage. | Marriage causes better health. | Better health causes lack of sadness. |
|  | Acidic diets cause the lack of calcium. | Calcium causes kidney stones | Kidney stones cause lack of energy. |
|  | Ice storms cause lack of electricity | Electricity causes injuries. | Injuries cause lack of mobility. |
| Cause¬ ◦ Cause¬ ◦ Cause | Aspirin causes lack of clotting. | Clotting causes lack of blood loss. | Blood loss causes shock. |
|  | Caffeine causes lack of sleep. | Sleep causes lack of studying. | Studying causes learning. |
|  | Warming causes the absence of ice. | Ice causes lack of traction. | Traction causes resistance to movement. |
|  | Dryness causes lack of vegetation. | Vegetation causes lack of erosion. | Erosion causes landslides. |
| Cause¬ ◦ Cause ◦ Cause | Alarms cause lack of burglaries. | Burglaries cause distress. | Distress causes suffering. |
|  | Antibiotics cause lack of infection. | Infections cause rashes. | Rashes cause blisters. |
|  | Doubt causes lack of joy. | Joy causes celebration. | Celebration causes happiness. |
|  | Sharks cause lack of swimming. | Swimming causes fatigue. | Fatigue causes slowness. |
| Cause ◦ Cause¬ ◦ Cause | Factories cause pollution. | Pollution causes lack of environmental health. | Environmental health causes ecological growth. |
|  | Fireworks cause explosions | Explosions cause lack of silence. | Silence causes calmness. |
|  | Medication causes drowsiness. | Drowsiness causes lack of driving. | Driving causes motion sickness. |
|  | Sanitation causes cleanliness. | Cleanliness causes lack of bacteria. | Bacteria cause disease. |
| Cause ◦ Cause ◦ Cause¬ | Conflict causes arguments. | Arguments cause problems. | Problems cause lack of friendship. |
|  | Exercise causes cardiovascular health. | Cardiovascular health causes fitness. | Fitness causes lack of weight gain. |
|  | Studying causes learning. | Learning causes intellectual development. | Intellectual development causes lack of ignorance. |
|  | War causes violence. | Violence causes suffering. | Suffering causes lack of peace. |
| Cause ◦ Prevent ◦ Prevent | Wind causes dryness. | Dryness prevents vegetation. | Vegetation prevents erosion. |
|  | Outsourcing causes competition. | Competition prevents inflation. | Inflation prevents revenues. |
|  | The sun’s energy causes storms. | Storms prevent travel. | Travel prevents stagnation. |
|  | Anxiety causes vigilance. | Vigilance prevents crime. | Crime prevents investment. |
| Prevent ◦ Cause ◦ Prevent | Encryption prevents identify theft. | Identify theft causes misery. | Misery prevents happiness. |
|  | Milk prevents diabetes. | Diabetes causes foot problems. | Foot problems prevent walking. |
|  | Rubber prevents slipping. | Slipping causes heat. | Heat prevents cooling. |
|  | Folic acid prevents heart defects. | Heart defects cause migraines. | Migraines prevent daily activities. |
| Prevent ◦ Prevent ◦ Cause | Green tea prevents Alzheimer's | Alzheimer's prevents remembering. | Remembering causes changes in the brain. |
|  | Exercise prevents arthritis. | Arthritis prevents walking. | Walking causes fat burning. |
|  | Time management prevents stress. | Stress prevents clear thinking. | Clear thinking causes clear language. |
|  | Dark chocolate prevents heart disease. | Heart disease prevents travel. | Travel causes jet lag. |
| Cause ◦ Prevent ◦ Cause¬ | Nervousness causes caution. | Caution prevents suffering. | Suffering causes the lack of selflessness. |
|  | Atheistic inaction causes theocracy. | Theocracy prevents secularism. | Secularism causes the lack of common social norms. |
|  | Cold weather causes ice. | Ice prevents swelling. | Swelling causes the lack of blood flow. |
|  | Melatonin causes sleep. | Sleep prevents fatigue. | Fatigue causes lack of focus. |
| Cause¬ ◦ Cause ◦ Prevent | Chemical dependence causes lack of productivity. | Productivity causes inflation. | Inflation prevents cut in interest rates. |
|  | Chemotherapy causes lack of appetite. | Appetite causes weight gain. | Weight gain prevents smoking cessation. |
|  | Laziness causes lack of work. | Work causes stress. | Stress prevents a long life. |
|  | Depression causes lack of motivation. | Motivation causes action. | Action prevents tragedy. |
| Prevent ◦ Cause¬ ◦ Cause | Bleach prevents algae. | Algae cause the lack of oxygen. | Oxygen causes rusting. |
|  | Alkaline diets prevent acid. | Acid causes the lack of calcium. | Calcium causes kidney stones. |
|  | Ice prevents swelling. | Swelling causes lack of blood flow. | Blood flow causes oxygenation. |
|  | Marriages prevents alcoholism. | Alcoholism causes lack of appetite. | Appetite causes indigestion. |
| Prevent ◦ Cause ◦ Cause | Planning ahead prevents stress. | Stress causes forgetfulness. | Forgetfulness causes trouble. |
|  | Vitamin B12 prevents nerve damage. | Nerve damage causes pain. | Pain causes lost workdays. |
|  | Exercise prevents arthritis. | Arthritis causes stiffness. | Stiffness causes impairment. |
|  | Tariffs prevent trade. | Trade causes economic development. | Economic development causes democratization. |
| Cause ◦ Prevent ◦ Cause | Spinning causes dizziness. | Dizziness prevents driving. | Driving causes traffic jams. |
|  | Throat irritation causes coughing. | Coughing prevents pneumonia. | Pneumonia causes inflammation. |
|  | Famine causes hunger. | Hunger prevents learning. | Learning causes brain growth. |
|  | Flooding causes road closures. | Road closures prevent vehicular traffic. | Vehicular traffic causes congestion. |
| Cause ◦ Cause ◦ Prevent | Plate tectonics cause earthquakes. | Earthquakes cause damage. | Damage prevents population recovery. |
|  | Garlic causes bad breath. | Bad breath causes embarrassment. | Embarrassment prevents social interaction. |
|  | Factories cause pollution. | Pollution causes global warming. | Global warming prevents snow fall. |
|  | Running causes endorphins. | Endorphins cause pleasure. | Pleasure prevents unhappiness. |
| Allow ◦ Prevent ◦ Prevent | Email allows communication. | Communication prevents misunderstanding. | Misunderstanding prevents reconciliation. |
|  | Buses allow travel. | Travel prevents stagnation. | Stagnation prevents change. |
|  | Transitioning allows pre-occupation. | Pre-occupation prevents paying attention. | Paying attention prevents accidents. |
|  | Communication allows proper planning. | Proper planning prevents poor performance. | Poor performance prevents realization of the plan. |
| Prevent ◦ Allow ◦ Prevent | Conflict prevents investment. | Investment allows expansion. | Expansion prevents overcrowding. |
|  | Entrenchment prevents innovation. | Innovation allows modernization. | Modernizations prevent flight delays. |
|  | Casts prevent bending. | Bending allows stretching. | Stretching prevents stiffness. |
|  | Video cameras prevent arrests. | Arrests allow immediate action. | Immediate action prevents possible harm. |
| Prevent ◦ Prevent ◦Allow | Olive oil prevents DNA damage. | DNA damage prevents cell division. | Cell division allows for renewal of the organism. |
|  | Protective eyewear prevents eye injuries. | Eye injuries prevent sports. | Sports allows competition. |
|  | Hand washing prevents infections. | Infections prevent healing. | Healing allows wholeness. |
|  | Protective wraps prevent corrosion. | Corrosion prevents heat dissipation. | Heat dissipation allows faster speeds. |
| Cause ◦ Prevent ◦ ¬Cause | Criminal investigations cause convictions. | Convictions prevent employment. | Lack of employment causes stress. |
|  | Failure causes discouragement. | Discouragement prevents motivation. | Lack of motivation causes underachievement. |
|  | Ice buildup causes dams. | Dams prevent flooding. | Absence of flooding causes river narrowing. |
|  | Thrombin causes clots. | Clots prevent blood flow. | Lack of blood flow causes heart attacks. |
| Prevent ◦ Cause ◦ ¬Cause | Flux prevents chemical reactions. | Chemical reactions cause electricity. | Lack of electricity causes inconvenience. |
|  | Realistic budgets prevent arguments. | Arguments cause stress. | Absence of stress causes muscle relaxation. |
|  | Tourism prevents unemployment. | Unemployment causes concern. | Lack of concern causes acceptance. |
|  | Insulation prevents evaporation. | Evaporation causes humidity. | Lack of humidity causes brittle hair. |
| Cause ◦ ¬Cause ◦ Prevent | Mass causes gravity. | Absence of gravity causes muscle atrophy. | Muscle atrophy prevents neuromuscular junctions. |
|  | Low pressure causes rainfall. | Lack of rainfall causes drought. | Drought prevents mosquito breeding. |
|  | Tobacco advertising causes smoking. | Absence of smoking causes weight gain. | Weight gain prevents extramarital affairs. |
|  | Cathode ray tubes cause light. | Absence of light causes darkness. | Darkness prevents photo aging. |

APPENDIX B

Calculus Approach to Defining Causal Relations and for Determining the Relative Frequency of Different Conclusions

In compositions leading to more than one conclusion, integral calculus can be used to determine the relative proportion of different conclusions. The first step in determining the relative frequency of different conclusions is to determine the overall area or volume of the distribution associated with a particular relation or composition of relations. Determining such an area is relatively simple in the case of HELP relations. In HELP relations, the affector and patient vectors both point toward the endstate, and their magnitudes do not constrain each other. For the sake of simplicity, let us assume the magnitudes can vary from 0 to 10.^[[1]](#footnote-1)^ Further, let us assume that the variable *x* corresponds to possible magnitudes of the affector vector and the variable *y*, to possible magnitudes of the patient vector. In general, we will assign the affector vector in the first premise to the innermost integral and the patient vector in the last premise to the outermost integral with vectors in intermediate premises assigned to intermediate integrals. In a HELP configuration every value of *x* can be paired with every value of *y*; hence of the possible combinations of *x* and *y* when plotted would form a square, as shown in the left panel of Figure B1, and since the area of a square is given by base times height, the total area would equal to 10^2^.


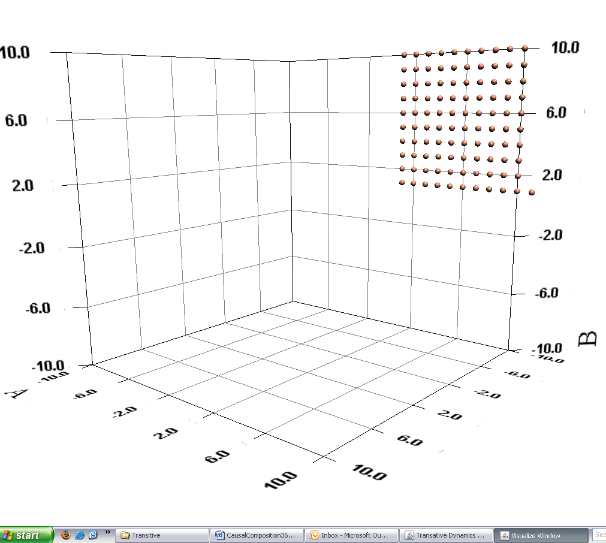

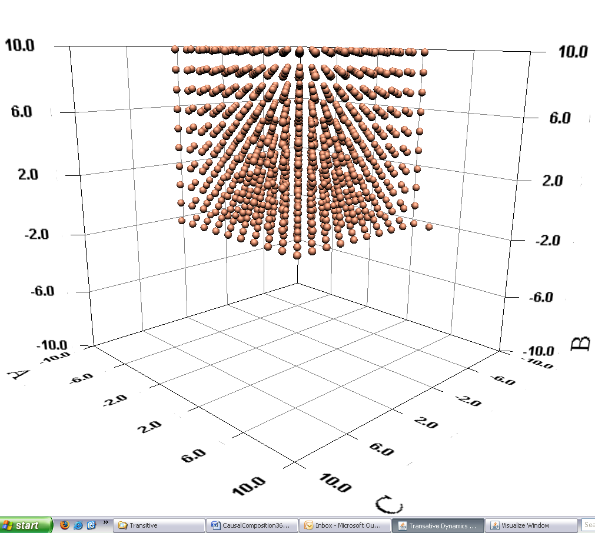


Figure B1. The scatter plot in the left panel shows possible combinations of magnitudes that the A and B vectors can take on in a HELP configuration. The scatter plot in the right panel shows the possible combinations of magnitudes that the A, B, and C vectors can take on in the composition of two HELP relations.

The same solution can be generated using double integration as shown in equation (1).

(1)

In order to solve the integral in (1), we first solve the innermost integral. The antiderivative of the implicit 1 in the innermost integral with respect to *x* is *x*. The solution to this integral is based on filling in the value of *x* with the upper value of 10 and then subtracting from it the value of *x* when it is assigned the lower value of 0. The solution, of course, would be 10, as worked out below.

Once the inner integral is solved, we can solve for the outer integral. The antiderivative of 10 with respect to *y* is 10*y*. We can solve the integral by filling in the value of *y* with the upper value of 10, giving 100, and then subtracting from it the value that results from filling in the *y* with 0. The result, of course, would be 100, as shown below.

As this example illustrates, the results from each integral are, in effect, multiplied together. Thus, whereas the representation of an individual configuration of forces involves vector addition, the representation of the distribution of the configurations associated with vector compositions is associated with vector multiplication.

Composing a HELP configuration with another HELP configuration would involve adding another vector. Again, if this vector is limited to values from 0 to 10, the resulting distribution of possible magnitude when plotted would form a cube with an area equal to 10^3^ (see right side of Figure B1). Calculating the volume in calculus involves adding an additional integral to the equation in (1), as shown in (2).

(2)

Composing three relations (e.g., HELP◦HELP◦HELP) requires at least four vectors leading to a hyper-volume. In calculus, the volume of the distribution could be calculated by including yet another integral. For each vector there must be a corresponding integral.

HELP configurations are simple because the magnitudes of the vectors in these configurations do not constrain each other. In contrast, in a CAUSE (or PREVENT) configuration the magnitude of the affector vector must always be greater than that of the patient vector. Thus, if we were to plot out the possible combinations of magnitudes associated with a single CAUSE configuration, they would fill in a region forming a triangle, or otherwise, for example, the area above the line defined by -*y*=*x* and the *x*-axis (see left side of Figure B2). The *y* variable is negative because it maps onto the patient vector, which points in the opposite direction from the endstate, which (in our example) points in a positive direction.


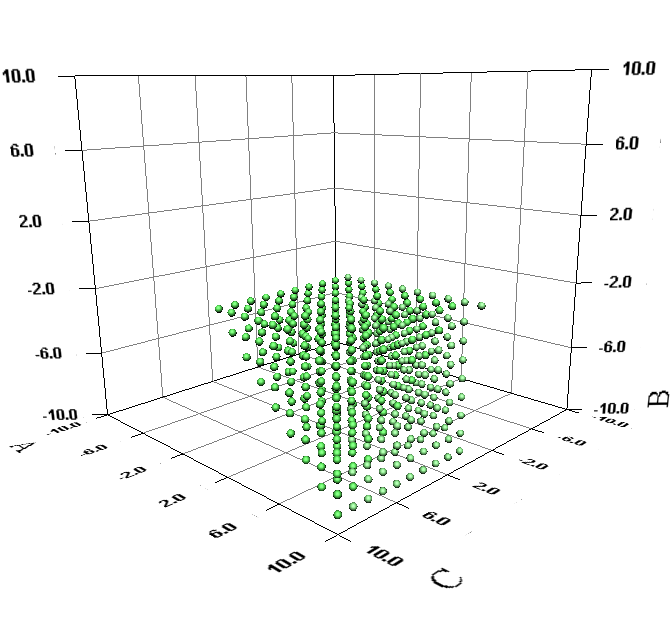

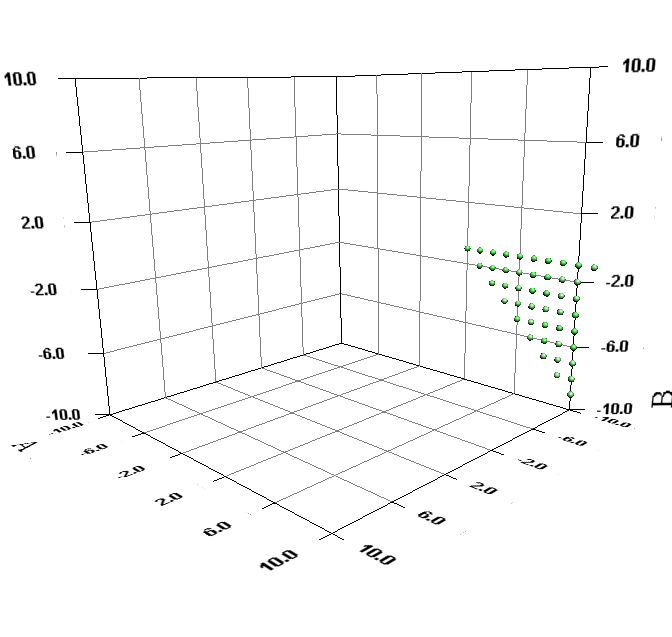


Figure B2. The scatter plot in the left panel shows possible combinations of magnitudes that the A and B vectors can take on in a CAUSE configuration. The scatter plot in the right panel shows the possible combinations of magnitudes that the A, B, and C vectors can take on in the composition of CAUSE and HELP relations.

Thus, assuming a region bounded by -*y*=*x*, in the case of a CAUSE relation, the *x* variable is constrained to range from 0 to -*y*. Such a constraint is easily factored into integration by limiting the upper and lower values of the integral, as demonstrated in the equation in (3). When the range is defined in terms of a variable, the variable is at a certain point treated as if it were a constant (as shown in step 3 below).

(3)

Notice that for the outer integral the values of the *y* variable range from -10 to 0; this is because the *y* variable corresponds to patient vector in the CAUSE relation, and the patient vector (in our example) points away from the endstate. Further note, because the values of *y* are always negative (except when at 0), the range of values in the *x* variable, which is defined in terms of the *y* variable, will always be positive since the range for the *x* variable is defined to occur between 0 and –*y*. Continuing with our integration, the antiderivative of -*y* is -½ *y*^2^, hence

Note that the area of the triangle is exactly half of the area of the square calculated from equation (1).

If a CAUSE configuration is composed with a HELP configuration, the resulting volume would be a triangle extruded along a dimension, and hence a prism. To calculate the volume of this prism (see right side of Figure B2), we simply add another integral to the equation in (3), giving us (4).

(4)


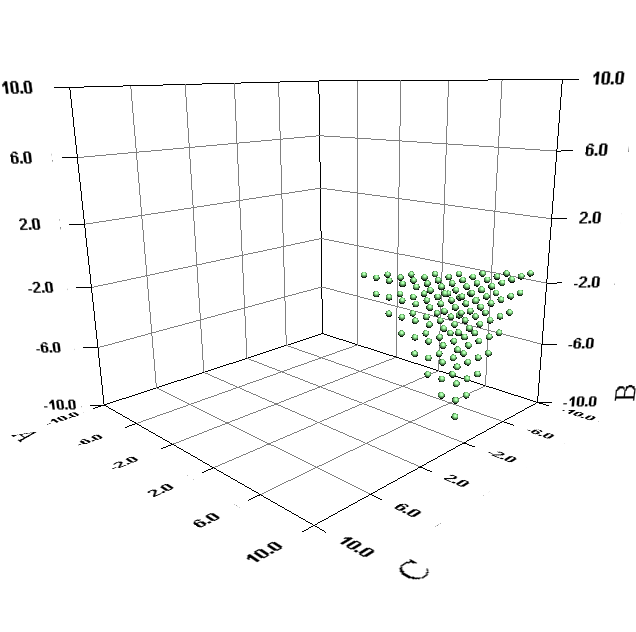
The volume entailed by equation (4) is equal to 500. If a CAUSE configuration is composed with another CAUSE configuration, the triangle associated with the original CAUSE would get smaller and smaller until it reached a point; thus, the resulting volume would be a tetrahedron (see Figure B3).

Figure B3. The scatter plot in the left panel shows the possible combinations of magnitudes that the A, B, and C vectors can take on in the composition of CAUSE and CAUSE.

The base triangle would get smaller and smaller because the possible magnitude of the vectors associated with each additional CAUSE would be constrained by the magnitudes of the earlier vectors. The equation in (5) captures the constraints that would be in place in the case of the composition CAUSE ◦ CAUSE depicted in Figure B3.

(5)

Interestingly, if the tetrahedron in Figure B3 were rotated 180° around the B-axis, its volume would, of course, be the same, but it would be associated with a different integral, namely the one shown in equation (6).

(6)

Notice that the only difference between equation (5) and (6) is that the *z* variable in the outermost integral ranges from -10 to 0 in equation (5), but from 0 to 10 in equation (6). The significance of this correspondence will soon become apparent.

*Computing the proportions associated with different conclusions*. As noted earlier, when a composition leads to more than one conclusion, we can use calculus to determine the proportion of the distribution associated with each type of conclusion. Consider again the composition CAUSE◦HELP. According to the force theory, this composition is consistent with both CAUSE and HELP conclusions. Equation (4) gives the overall volume of the distribution associated with CAUSE◦HELP. In order to determine the proportion of this distribution associated with each conclusion, we need to partition the distribution into two parts. This can be done by splitting the range of values in the middle integral in equation (4). In order to calculate the volume associated with CAUSE, we limit the values of *y* to values from 0 to -*z*, as shown in equation (7).

(7)

Note that the equation in (7) is exactly the same as the equation in (6), confirming that this part of the CAUSE◦HELP distribution would be that associated with CAUSE; when we compute the volume associated with equation (7), the result is 166. As noted earlier, the volume of the entire CAUSE◦HELP distribution is 500. Hence, the proportion of CAUSE conclusions in a CAUSE◦HELP distribution would 166/500, that is, 33 percent. In order to calculate the volume of the distribution associated with HELP, we integrate over the range of values in equation (4) that were not covered in equation (7); specifically we integrate from -*z* to -10, as shown in (8).

(8)

The solution to equation (8) is 333. Dividing this number by the total volume, 500, gives .666, or 67 percent. Of course, we could have reached the same result by subtracting the proportion associated with CAUSE from 1. It should be clear that calculating the relative proportions does not depend on the specific limits that are chosen. Instead of 10, we could have chosen 13, 82, or 100; the volumes would differ, but the ratios of the volumes associated with different conclusions and the volume of the entire distribution would be the same.

A third way to calculate these proportions is to use a simulation process. Such a process has been implemented in the following program: <http://userwww.service.emory.edu/~pwolff/Transitivedynamics.htm>. The program allows users to construct compositions. A simulation identifies all of the combinations of magnitudes that are compatible with the particular relations in the composition. As the magnitudes are varied, the program tallies the different kinds of conclusions that are generated, which are then used to calculate the proportion of different kinds of conclusions. The simulation process requires that the magnitudes of the vectors be limited to an upper range (determined in the program by the length of the endstate vector in the first premise). Increasing the size of this limit, in effect, increases the number of boxes under the distribution, thereby improving the approximation. The effect of increasing the limit can be observed in the case of the CAUSE◦HELP composition. A limit of 5 results in CAUSE 41%, HELP 59%; a limit of 10 results in CAUSE 37%, HELP 63%; a limit of 20 results in CAUSE 35%, HELP 65%; a limit of 100 results in CAUSE 34%, HELP 66%; and a limit of 500 results in CAUSE 33%, HELP 66%. Thus, the percentage of CAUSE and HELP conclusions converges on the exact proportions determined in calculus as the size of the limit is increased. In effect, then, the program sets up and solves the integrations entailed by various compositions.

1. According to the force theory, the values of each vector in a HELP configuration must be greater than 0, but assuming a value of 0 will have no consequences for determining the relative size of the different parts of the distribution and makes the calculations easier to compute. Similarly, the upper end of the range can be set to any number, so long as it is greater than the number in the lower range, but here we choose 10 for ease of computation. [↑](#footnote-ref-1)
